# Replacing Soybean Meal with Hemp Leaves in a Dairy Cow Diet: Plasma Antioxidative Capacity, Inflammatory Parameters and Milk Constituents

**DOI:** 10.3390/ani15101414

**Published:** 2025-05-14

**Authors:** Jessica Schwerdtfeger, Solvig Görs, Dirk Dannenberger, Björn Kuhla

**Affiliations:** Research Institute for Farm Animal Biology (FBN), Wilhelm-Stahl-Allee 2, 18196 Dummerstorf, Germany; schwerdtfeger@fbn-dummerstorf.de (J.S.); goers@fbn-dummerstorf.de (S.G.); dannenberger@fbn-dummerstorf.de (D.D.)

**Keywords:** oxidative stress, pro-inflammatory cytokines, amino acids, Santhica 27, cannabinoids, PUFA, lipid peroxidation

## Abstract

Hemp is rich in bioactive compounds known to exert antioxidant and anti-inflammatory properties. We hypothesised that feeding industrial hemp leaves would increase the antioxidative capacity, reduce pro-inflammatory markers, and modulate the milk amino and fatty acid profiles of dairy cows. Twelve Holstein dairy cows were randomly assigned to a total mixed ration (TMR) containing 7.4% industrial hemp leaves (HEMP) of the Santhica 27 variety or a TMR-based diet containing 3.5% soybean meal (CON). Diets were fed in a cross-over design, each for 2 weeks divided by a 2-week washout period. HEMP cows revealed higher plasma and milk cannabinoid concentrations, plasma antioxidative capacity and anserine concentration, but lower plasma π-methylhistidine, α-aminoadipic acid and isoleucine concentrations. Lipid peroxidation and tumour necrosis factor-alpha concentrations were not different between groups. The leukocyte RELA mRNA expression was higher, whereas the TNFA mRNA abundance tended to be lower in the HEMP group. In milk, n-3 polyunsaturated fatty acid (PUFA) concentrations were higher, and the n-6/n-3 PUFA ratio was lower in HEMP than in CON cows. The results provide evidence that the supplementation with Santhica 27 hemp leaves increases the antioxidative capacity of dairy cows and results in a transfer of cannabinoids into milk.

## Simple Summary

We studied if feeding dried industrial hemp leaves containing very low tetrahydrocannabinol concentrations but substantial amounts of antioxidants such as phenols, tannins and flavonoids would increase the antioxidative capacity and reduce pro-inflammatory and oxidative stress markers of dairy cows. The results show that 7.4% supplementation of dried industrial hemp leaves of the variety Santhica 27 as compared to a soybean meal-containing diet increases the plasma total antioxidative capacity, the concentration of the antioxidant anserine on the expense of π-methylhistidine (the precursor of the former), the mRNA expression of a key regulator controlling cytokine expression of white blood cells, as well as the milk fatty acid n-6/n-3 ratio of dairy cows. The mRNA abundance of a pro-inflammatory cytokine tended to be reduced after hemp feeding. Thus, feeding cows Santhica 27 leaves may prevent them from oxidative stress and inflammation.

## 1. Introduction

Hemp is one of the oldest cultivated plants in the world, as many parts of the plant can be used in a variety of applications. Currently, the stalks are deployed for textile and industrial fibre production; the seeds can be used for food production, and the flowers for the extraction of cannabinoids. The leaves of hemp plants can used to prepare tea but also fed to various animal species [1,2,3].

Due to its noteworthy crude fibre and crude protein contents [4,5], leaves attached to the whole aboveground industrial hemp plant have recently been tested as feed in dairy cattle nutrition [6,7,8]. The inclusion of 13% (on a DM basis) spent hemp biomass (SHB), the whole plant residue remaining after alcoholic extraction, or 7.4% dried hemp leaves in the ration of dairy cows decreased dry matter intake (DMI) but not dry matter (DM) digestibility [7].

Apart from macronutrients, hemp leaves contain several bioactive, ethanol-soluble cannabinoids, and many of them modulate oxidative stress, inhibit the release of excitotoxic amino acids and cytokines, or exert anti-inflammatory properties [9,10,11]. The anti-inflammatory properties are, among others, attributable to the influence of cannabinoids, such as tetrahydrocannabinol (THC), cannabidiol (CBD), and cannabigerol (CBG). These cannabinoids can affect cellular signalling pathways, including the phosphorylation of NF-κB and the subsequent inhibition of the transcription of pro-inflammatory cytokines such as interleukin-1beta (IL-1ß) and tumour necrosis factor alpha (TNF-α) [12,13].

Although the total CBD concentration of SHB is much lower than in non-extracted hemp plants, the inclusion of 6 to 11% SHB in the diet of dairy cows reduced their plasma IL-1ß but not TNF-α concentrations [14]. However, previous research showed that cannabinoids can be transferred into the milk and can exceed the acute THC (tetrahydrocannabinol) reference dose for humans. For example, feeding a 0.92 kg of DM hemp silage containing 58.3 mg/kg of DM ∆9-THC resulted in the exceedance of the acute reference dose by a factor of 1.5 in infants with high milk consumption, whereas feeding 1.68 kg hemp silage per cow containing 1254.7 mg/kg of DM ∆^9^-THC led to the acute reference dose being exceeded in all consumer groups [6]. Furthermore, feeding industrial hemp silage to dairy cows, even in small amounts, altered animal behaviour and impaired animal health [6]. In order to still make use of the positive effects of non-THC cannabinoids, only hemp varieties containing no or very low THC concentrations (e.g., 1/100 of the threshold of 0.3% of DM, valid in the European Union) come into question as nutritional supplements. The variety Santhica 27 contains particularly low THC concentrations [15] and thus may serve as a feed supplement with potential antioxidant, anti-inflammatory and amino acid-modulating effects in dairy cattle.

In addition to cannabinoids, industrial hemp leaves are a source of phenolic and flavonoid compounds, some of them being soluble in ethanol. Phenolic and flavonoid compounds exhibit antioxidant properties, which could improve the antioxidant capacity of cows alike [16]. However, feeding SHB to ruminants did not improve the antioxidant capacity in plasma, the activity of enzymes combating oxidative stress or parameters related to immune function [7,14,17], likely because only limited amounts of phenolic, flavonoids and further biologically active substances remain in SHB after extraction. Thus, the effect of feeding industrial hemp leaves with a very low THC content on the antioxidative capacity of dairy cows still remains to be investigated.

The SHB, similar to soya, further contains minor n-3 but even more n-6 polyunsaturated fatty acids (PUFA) concentrations with an n-6/n-3 ratio of about 6:1 [7]. When SHB was supplemented to a total mixed ration (TMR), resulting in an n-6/n-3 ratio of 2.4:1, the percentage of milk *C*18:0 and the percentage of de novo milk fatty acids were significantly reduced, which led to improved nutritional indices of the milk for human nutrition, e.g., the hypocholesterolemic/hypercholesterolemic (h/H) index or atherogenic index (AI) [7]. Yet, it remains to be elucidated how the feeding of THC-free industrial hemp leaves, not depleted of lipids, affects milk fatty acid concentrations and nutritional indices.

The aim of this study was, therefore, to investigate the effects of feeding dried Santhica 27 leaves compared to soybean meal on performance traits, the antioxidant capacity and inflammatory parameters, plasma cannabinoid and amino acid concentrations, as well as milk fatty and amino acid profiles of dairy cows. We hypothesised that the supplementation of dried Santhica 27 hemp leaves to the diet of dairy cows would improve the antioxidant capacity, reduce the abundance of excitatory amino acids and pro-inflammatory cytokines, and modulate the milk amino and fatty acid profiles.

## 2. Materials and Methods

### 2.1. Ethical Considerations

The experimental protocol was evaluated by the ethical committee and approved by the Federal Office of Agriculture, Food Security and Fishery Mecklenburg—Western Pomerania, Rostock, Germany (LALLF, permission no. 7221.3-1-028/22) and conducted in accordance with the ARRIVE guidelines (https://arriveguidelines.org/), the European Directive 2010/63/EU and the German Animal Welfare Act. Persons involved in sample preparation and analyses were blinded. Persons who collected blood samples, prepared the feed, and fed the cows were not blinded.

### 2.2. Animals and Experimental Design

For this study, 12 first-lactating German Holstein dairy cows were randomly selected from the herd of the Experimental Farm for Cattle (FBN, Dummerstorf, Germany) and fed two isoenergetic and isonitrogenous formulated diets in a cross-over design. The experimental design consisted of 3 blocks of 4 animals each.

At the beginning of the experimental trial, cows were 227 ± 29 days in milk (DIM) and 116 ± 45 days in gestation (mean ± SD). In experimental period 1, cows were fed ad libitum a TMR supplemented with 7.4% (on dry matter (DM) basis) dried industrial hemp leaves (HEMP) of the variety “Santhica 27” containing 154.17 g/kg of DM crude protein and 11.45 MJ/kg of DM metabolisable energy (ME) or a TMR containing 3.5% soybean meal (CON) containing 152.17 g/kg of DM crude protein and 11.45 MJ/kg of DM ME for 21 days. During the 2-week washout period, all cows were fed a hemp- and soy-free TMR. In experimental period 2, cows were fed the opposite diet for ad libitum intake for a further 21 days. All cows had ad libitum access to feed and water throughout the trail. Cows were fed at 0730 h and 1430 h.

Groups were housed in the free-ranging barn of the experimental facilities at FBN to measure the individual daily feed intake using the Roughage Intake Control system (RIC, Insentec B.V., Repelweg, The Netherlands) in the first two weeks of each experimental period. Feed samples were taken twice a week, dried at 60 °C for 24 h, ground (1 mm particle size) and further dried at 103 °C for 4 h to determine feed DM according to the methodological guidelines of the Association of German Agricultural Analytic and Research Institutes (VDLUFA) [18]. Dried feed samples were sent to the Landwirtschaftliche Untersuchungs- und Forschungsanstalt (LUFA GmbH, Rostock, Germany) for the analyses of nutrient composition, including crude ash, crude protein, crude fat, crude fibre, acid detergent fibre based on organic matter basis, neutral detergent fibre based on organic matter basis and starch. Based on the analysis results, the metabolisable energy (ME) of feed was calculated according to the Society of Nutrition Physiology (GfE, 2001) [19]:

ME MJkgDM=6.0756+0.19123∗ EE+0.02459∗ CP−0.000038∗ CF2−0.002139∗ EE2−0.00006∗ CP2,

including ether extract (EE), crude protein (CP) and crude fibre (CF) in g/kg of DM of feed.

Cows were milked, and milk yield was recorded with a tandem milking parlour equipped with a milk meter (DeLaval GmbH, Glinde, Germany) at 0630 h and 1700 h. Twice a week, a pooled milk sample of the evening and the subsequent morning milking was taken in the first two weeks of each experimental period. Milk constitutes were analysed by infrared spectroscopy, and the somatic cells were counted fluoro-optically at the State Inspection Association for Performance and Quality Testing Mecklenburg—Western Pomerania e.V. (LKV Güstrow, Germany). The energy-corrected milk yield (ECM) was calculated using the following equation:

ECM=kg milk yield∗((0.38∗%Fat+0.21∗%Protein+1.05)/3.28)

The body weight (BW) of the cows was determined after each milking using a walk-through scale. The average BW and the average metabolic BW (mBW = BW^0.75^) were calculated for the first two weeks of each experimental period. The rectal temperature was measured daily before the morning feeding to monitor animal health. Blood samples were drawn from the jugular vein at 0700 h in 9 mL ethylenediaminetetraacetic acid (EDTA)-containing tubes (Sarstedt, Nümbrecht, Germany) on days 0, 7 and 14. The obtained blood samples were immediately placed on ice and subsequently centrifuged at 1.570× *g* and 4 °C for 20 min. The plasma was separated, the buffy coat was mixed with lysis buffer DL (MACHEREY-NAGEL, Düren, Germany), and samples were stored at −80 °C until further analysis.

### 2.3. Amino Acid Analysis

For the free amino acid analysis in whey, milk samples were centrifuged twice at 50,000× *g* and 4 °C for 20 min, and the obtained clear whey was diluted fivefold with ultrapure water. Plasma samples were tenfold diluted with ultrapure water.

The amino acid analysis was performed as described by Krömer (2006) [20]. In brief, total amino acids in plasma and whey were separated after pre-column derivatisation with ortho-phthalaldehyde/3-mercaptopropionic acid (primary amino acids) and 9-fluorenylmethoxycarbonyl chloride (secondary amino acids) and prior blocking of SH groups with iodoacetic acid after cleavage of sulphur bridges with 3-mercaptopropionic acid using a high-performance liquid chromatography (HPLC) column at 40 °C (250 × 4.6 mm Gemini^®^ 5 µm *C*18 110 Å with 4 × 3 mm pre-column (both Phenomenex, Aschaffenburg, Germany) and detected by a fluorescence detector.

Chromatographic separation was performed using a gradient of 40 mM phosphate buffer (pH 7.45) and acetonitrile/methanol/water (45:45:10) ranging from 7 to 100% at a flow rate of 0.8 mL/min in 48 min. Ortho-phthalaldehyde derivatives were detected at 340 nm excitation and 450 nm emission, and the 9-fluorenylmethoxycarbonyl derivatives at 266 nm excitation and 305 nm emission. All amino acids and their metabolites were quantified against multipoint calibrated external standards.

### 2.4. Fatty Acids Analysis

Fatty acid analysis of the diets was performed according to the procedure recently described by Tadesse et al. [21]. The feed samples were finely ground to 1 mm, 2 g weighed in 10 mL screw-capped Pyrex tubes, and 2.0 mL of nonadecanoic acid (4.0 mg) was added as an internal standard. For fatty acid extraction, 3 mL of 5% methanolic hydrogen chloride (HCl) was added and vortexed in tightly closed Pyrex tubes for 2 h at 60 °C in a water bath. After cooling, the sample solution was treated with 10 mL 6% potassium carbonate (K_2_CO_3_) solution and vortexed. The solutions were centrifuged at 4 °C, 1200× *g* for 5 min, and finally, the fatty acid methyl esters (FAMEs) were extracted two times with 2 mL of n-hexane. After being dried with 1 g sodium sulphate and cleaned with activated charcoal of the organic phase as required, the extracts were filtrated and evaporated using a vacuum centrifuge at 438× *g*, 30 °C, 30 min. Finally, the extracts were stored at −18 °C until gas chromatography (GC) analysis.

Fatty acids were extracted using a fatty acid extraction kit (MAK174, low standard, Sigma-Aldrich, St. Louis, MO, USA). According to the kit protocol, the milk samples were stirred, and 200 mg milk was treated with 3 mL extraction solvent consisting of chloroform/methanol (2:1, vol/vol) containing the ethyl ester of nonadecanoic acid (C19:0) as internal standard.

After vortexing and a reaction time of 4 min, 0.5 mL of an aqueous buffer (kit buffer solution) was added, and the sample was vortexed again. Subsequently, the milk extraction solution was transferred into a syringe system containing a filter (provided in the kit). Finally, the eluted solvent contained the chloroform phase with total milk lipids. To complete the extraction, the solutions were incubated overnight at 4 °C.

Then, the solvents of the extracted lipids were evaporated with a gentle stream of nitrogen, and the amounts of total lipids were weighed. The milk lipid extracts were dissolved in toluene for methyl ester derivatisation. Next, 0.5 M sodium methoxide in methanol was added to the samples, which were shaken in a 60 °C water bath for 10 min.

Subsequently, 14% boron trifluoride in methanol was added to the mixture, which was then shaken for an additional 10 min at 60 °C. The fatty acid methyl esters (FAMEs) were extracted three times in 2 mL of n-hexane. The FAMEs were stored at −18 °C until used for GC analysis.

The GC analysis of fatty acids in feed and milk samples was performed as described before [22]. Briefly, separation and quantification of the fatty acid methyl esters were conducted using a fused silica capillary column (100 m × 0.25 mm, Agilent, Santa Clara, CA, USA) on a PerkinElmer Clarus 680 gas chromatograph equipped with an autosampler and flame ionisation detector. For calibration, the methyl esters of *cis*-vaccenic acid (*C*18:1*cis*-11), docosapentaenoic acid (*C*22:5n-3), rumenic acid (*C*18:2*cis*-9, trans-11), docosatetraenoic acid (*C*22:4n-6) and stearidonic acid (*C*18:4n-3) of the “Sigma FAME” standard were used. The five-point calibration of single fatty acids ranged from 16 to 415 µg/mL.

To evaluate the nutritional value of milk for human nutrition, the thrombogenic index (TI) and atherogenic index (AI) were calculated according to Irawan et al. (2024) [7] using the following equations:

$$\begin{array}{c} TI=(C14:0+C16:0+C18:0)/[(0.5*\Sigma MUFA)+(0.5\times \Sigma n-6PUFA)\\ \;+(3\times \Sigma n-3PUFA)+(n-3/n-6)] \end{array}$$

AI=[C12:0+(4∗C14:0)+C16:0]/ΣUFA,

in which *C*12:0 is lauric acid, *C*14:0 is myristic acid, *C*16:0 is palmitic acid, *C*18:0 is stearic acid*,* UFA is unsaturated fatty acids, MUFA is monounsaturated fatty acids, and PUFA is polyunsaturated fatty acid.

In addition, the hypocholesterolemic/hypercholesterolemic ratio (h/H) was calculated according to Chen and Liu (2020) [23]:

h/H=(cis−C18:1+ΣPUFA)/(C12:0+C14:0+C16:0),

in which *cis*-*C*18:1 is oleic acid, *C*12:0 is lauric acid, *C*14:0 is myristic acid, *C*16:0 is palmitic acid and PUFA is polyunsaturated fatty acid.

Furthermore, the health-promoting index (HPI) was calculated according to Chen et al. (2004) [24] as follows:

HPI=ΣUFA/[C12:0+(4∗C14:0)+C16:0],

in which *C*12:0 is lauric acid, *C*14:0 is myristic acid, *C*16:0 is palmitic acid and UFAs are unsaturated fatty acids.

### 2.5. Analysis of Secondary Plant Metabolites

For the analysis of total tannins, condensed tannins, and total phenol concentrations, an extract was prepared from the feedstuff according to Cork and Krockenberger (1990) with some changes [25]. Briefly, 50 mg of dried and ground (0.7 mm) TMR or individual feed components was mixed with 10 mL 50% acetone and incubated on the shaker for 24 h. Subsequently, the extract was centrifuged at 2500× *g* and 4 °C for 15 min.

The total phenol and total tannin concentrations were determined as described by the Joint FAO/IAEA Division of Nuclear Techniques in Food and Agriculture with some modifications [26]. In brief, 0.5 mL of the supernatant was mixed with 0.25 mL Follin–Ciocalteau reagent (1:1 diluted with aqua dest. Carl Roth, Karlsruhe, Germany) and incubated for 1 min at room temperature. Subsequently, 1.25 mL of 20% Na_2_CO_3_ was added. The mixture was vortexed and incubated for 40 min at room temperature in the dark. The absorption was measured at 700 nm wavelength. Tannic acid (Merck, Darmstadt, Germany) was used to create a standard curve ranging from 0 to 10 µg.

For the analysis of total tannin concentrations, 100 mg polyvinylpyrrolidone (Merck, Darmstadt, Germany) was mixed with 1 mL distilled water and 1 mL of the acetonic feed extract (1:10 diluted with water). The mixture was vortexed and incubated at 4 °C for 15 min. After additional vortex, the mixture was centrifuged at 2.500× *g* for 15 min. Subsequently, 0.5 mL of the supernatant was mixed with 0.25 mL Follin–Ciocalteau reagent (1:1 diluted in water) and incubated for 1 min at room temperature. After adding 1.25 mL of 20% Na_2_CO_3_, the mixture was vortexed and incubated for 40 min in the dark. Subsequently, the mixture was centrifuged at 16,100× *g* for 5 min at room temperature, and the absorbance was measured at 700 nm. To calculate the total tannin content, the measured absorbance was subtracted from the absorbance of the total phenol content measurement (see above).

Condensed tannin concentrations were analysed according to Porter et al. (1986) and recommended previously [26,27]. In brief, for the analysis of the condensed tannin concentration, 100 µL of the acetonic feed extract was mixed with 600 µL butanol–HCl reagent (butanol–HCl 95:5 *v*/*v*) and 20 µL ferric reagent (2% ferric ammonium sulphate in 2N HCl). The mixture was vortexed and incubated at 70 °C for 4 h. After cooling, the absorbance was measured at 550 nm wavelength. Samples for the determination of total tannin, condensed tannin, and total phenol concentrations were analysed in triplicate. The intra-assay coefficient of variation (CV) was 8.6% for the analyses of condensed tannin in TMR samples and 5.9% for individual feed component samples. For the analyses of the total phenol concentration, intra-assay CV was 5.9% for individual feed component samples and 4.7% for TMR samples. The intra-assay CV was 4.1% for the analyses of total tannin in TMR samples and 1.1% for individual feed component samples.

For the measurement of total flavonoid concentration in TMR, hemp leaves, straw, rapeseed and soybean meal, approximately 1 g of each sample was incubated with 30 mL 75% ethanol in an ultrasonic bath at 60 °C for 30 min. The obtained extract was filtered using a paper filter (MN 614, MACHEREY-NAGEL, Düren, Germany), and the extraction procedure was repeated twice. The combined extracts were diluted with 75% ethanol to 100 mL stored at 4 °C until further analysis.

The total flavonoid concentration was determined using quercetin as standard as described by Ruiz-Reyes et al. (2022) [28] with the following modifications: A mixture of 200 μL feed extract, 0.8 mL 70% ethanol, and 1 mL aluminium chloride (AlCl_3_) solution (20 mg AlCl_3_ in 1 mL 70% ethanol and 5% acetic acid) was incubated in the dark for 30 min and subsequently measured at a wavelength of 410 nm using a microplate reader (Sunrise, Tecan Trading, Switzerland). All samples were run in duplicate. Intra-assay CV was 8.5%.

The concentrations of the cannabinoids cannabidivarin (CBDV), cannabidiol (CBD), cannabinol (CBN), Δ9-tetrahydrocannabivarin (Δ9-THCV), Δ8-tetrahydrocannabivarin (Δ8-THC), Δ9-tetrahydrocannabinol (Δ9-THC) and tetrahydrocannabinolic acid (THCA) in Santhica 27 hemp leaves were analysed by the Chemical and Veterinary Federal Office (Münsterland-Emsche-Lippe, Germany) using LC-MS/MS as described by Wagner et al. (2022) [6]. The detection limit was 0.5 µg/kg and the limit of quantification was 1 µg/kg. The recovery rates ranged between 70 and 120% and were corrected via isotope-labelled ISTs. The expanded measurement uncertainty was 25%. The specificity was verified via the retention time and specific mass transitions.

For the cannabinoid analyses, 0.5 mL of plasma or milk was mixed with the internal standard DHA-ethanolamide-d4 and acidified with citrate buffer. The samples were extracted twice with ethyl acetate and dried under a nitrogen stream. The resulting extract was dissolved in 100 µL methanol/water (60:40), including 1 µg/mL butylated hydroxy toluene. The cannabinoid concentrations in plasma and milk, namely Δ9-THC, THCA, CBD, CBN, cannabichromene (CBC), CBG, cannabigerolic acid (CBGA), and cannabidiolic acid (CBDA) using an HPLC coupled to a triple-quad mass spectrometer with electrospray ionisation (Agilent 6495/1290, Santa Clara, CA, USA) equipped with a Poroshell E*C*120 column (100 × 2.1 mm, 2.6 µm, Agilent, Santa Clara, CA, USA). Separation was performed using a gradient of 5 mM aqueous ammonium acetate solution and methanol. Cannabinoids were identified on the basis of at least three characteristic mass transitions and the retention time. Authentic standards (Sigma-Aldrich, St. Louis, MO, USA) in relation to the internal standard were used for the quantification. The calibration curves were linear in the range of 0.01 to 10 ng/mL (r^2^ > 0.99). Sample preparation and concentration measurement of the cannabinoids were performed by Lipidomix GmbH (Berlin, Germany). The detection limit was 0.003 ng/mL, and the limit of quantification was 0.01 ng/mL. The extraction yields ranged between 80 and 110% and were taken into account in the calculations.

### 2.6. Determination of the Antioxidative Capacity

To measure the antioxidant capacity of the feed, an extract from a 1 g sample was prepared as described for the measurement of total flavonoid concentration. For the determination of the ferric-reducing antioxidant power (FRAP), a working reagent (0.3 M acetate buffer, 0.01 M 2,4,6-Tri(2-pyridyl)-1,3,5-triazine (TPTZ) in 0.04 M HCl and 0.02 M FeCl_3_ × 6H_2_O, 10:1:1 *v*/*v*/*v*) was prepared as described by Wojtunik-Kulesza (2020) [29]. For the analysis of FRAP, 10 μL of the feed extract was mixed with 90 μL working reagent solution, and the absorbance was measured at 595 nm at room temperature after 10 min. Analyses were run in duplicate. The intra-assay CV was 9.6% for TMR samples and 2.3% for individual feed component samples.

The ferric-reducing ability of plasma was determined as described by Benzie and Strain (1996) with minor modifications [30]. After thawing on ice, 100 µL of plasma was mixed with 100 µL 75% ethanol and centrifuged at 13,000× *g* for 10 min, while rumen fluid was centrifuged without prior ethanol addition. Ten microlitres of the respective supernatant was mixed with ninety microlitres of FRAP working reagent, and the absorbance was measured as described above. Analyses were run in duplicate. The intra-assay CV was 2.5%.

To analyse FRAP in whey, milk samples were thawed, and 1 mL was centrifuged at 50,380× *g* for 20 min at 4 °C. Subsequently, 400 µL of the aqueous phase was taken and mixed with 75% ethanol and again centrifuged. Ten microlitres of the supernatant was mixed with 90 microlitres of the FRAP working reagent, and the absorbance was measured as described above. Analyses were run in duplicate. The intra-assay CV was 3.9%.

In addition, a 2,2′-azino-bis(3-ethylbenzothiazoline-6-sulfonic acid) (ABTS) decolourisation assay was used to assess antioxidant capacity in feed and plasma adapted from Re et al. (1999) with some modifications [31]. In brief, the ABTS stock solution was prepared containing 900 µM ABTS (Merck, Darmstadt, Germany) and 450 µM potassium persulfate. Plasma samples were thawed on ice and 1:3 diluted with phosphate-buffered saline +0.5 mM EDTA. Ten microlitres of the respective feed extract or diluted plasma samples was mixed with two hundred microlitres of ABTS stock solution, and the absorbance was measured at 700 nm at room temperature after 5 min incubation in the dark. A Trolox solution diluted with ethanol was used to create a standard curve between 0 and 1200 µM. Plasma samples were run in triplicate, and feed samples in duplicate. Intra-assay CV was 2.0% for plasma samples, 0.7% for individual feed components and 0.4% for TMR samples.

The antioxidant activity in feed samples was further assessed by a 2,2-diphenyl-1-picrylhydrazyl (DPPH) assay, according to Brand-Williams et al. (1995) [32]. Briefly, 50 µL of the feed extract was mixed with 100 µM DPPH in ethanol and incubated for 30 min in the dark. Absorbance was measured at 520 nm wavelength. All DPPH analyses were run in triplicate. The intra-assay CV was 5.0% for TMR samples and 5.1% for individual feed components. The antioxidant capacity determined by ABTS and DPPH assays is expressed as Trolox-equivalent antioxidant capacity (TEAC).

### 2.7. Analysis of Thiobarbituric Acid Reactive Substances

The thiobarbituric acid reactive substances (TBARS) in plasma samples obtained on day 14 were determined using a TBARS Parameter Assay Kit (KGE013, R&D Systems Europe, Abingdon, United Kingdom) according to the manufacturer’s instructions. Absorbance was measured using a microplate reader (Sunrise, Tecan Trading, Männedorf, Switzerland). Samples were analysed in duplicates. The intra-assay CV was 4.5%.

### 2.8. Gene Expression in White Blood Cells

Total RNA was extracted from 400 µL buffy coat using the NucleoSpin RNA Blood Kit (MACHEREY-NAGEL, Düren, Germany) according to the manufacturer’s instructions. The RNA quality was assessed using an Agilent 2100 Bioanalyzer (Agilent Technologies Inc., Santa Clara, CA, USA). RNA integrity numbers (RIN) between 5 and 9.4 (mean: 7.7, SD: 1.1) were yielded. The cDNA synthesis and qPCR were performed as described recently with the following modification [33].

The PCR contained 2 µL cDNA (10 ng/µL), 1 µL H_2_O, 0.5 µL of each primer (4 µM), and 6 µL 2 × Puffer SensiFAST SYBR No-ROX mix (Meridian Bioscience, Cincinnati, OH, USA). Data were quantified using qbase software (Biogazelle, Gent, Belgium).

Tyrosine 3-monooxygenase/tryptophan 5-monooxygenase activation *(YWHAZ)*, ribosomal protein S9 (*RPS9*), and succinate dehydrogenase complex flavoprotein subunit A1 (*SDHA*) were used as reference genes (M-value: 0.298, CV-value: 0.119). The primer sequences are shown in Supplementary Table S1.

The efficiency of amplification was calculated using LinReg-PCR software (v.2014.4, Academic Medical Centre, Amsterdam, The Netherlands). Only 6 samples per group could be analysed because the RNA concentrations of the remaining samples were insufficient.

### 2.9. Tumour Necrosis Factor Alpha Concentrations

Plasma tumour necrosis factor alpha (TNF-α) concentration in samples obtained on day 14 of each period was measured using a bovine ELISA kit (Bovine TNF Alpha ELISA Kit—LS-F5014, LifeSpan BioScience, Washington, DC, USA) in accordance with the manufacturer’s instructions. Absorbance was measured using a microplate reader (Sunrise, Tecan Trading, Männedorf, Switzerland). All samples were analysed in duplicates. The intra-assay CV was 5.6%. Three samples (2 HEMP, 1 CON) had to be excluded from the statistical analysis because the CV exceeded the cut-off value of 15% that was set prior to the experiment.

### 2.10. Statistical Analysis

The required sample size to demonstrate the equivalence of parameters was calculated using ‘sampleN. TOST()’ from the “PowerTOST” package [34] in R Statistical Software (version 4.4.2) [35]. A coefficient of variation of 15%, a ratio of the means of 0.9, a lower and upper equivalence limit of 0.75 and 1.33, respectively, and a probability for the type I error of α = 0.05 and for the type II error of β = 0.2 were assumed. Additionally, assuming Cohen’s d = 1.0 and a type I error probability of α = 0.05 and power = 0.8, the sample size to detect differences between two parameters was calculated using the function pwr.t.test()’ from the R package ‘pwr’ [36].

Statistical analysis was performed using R Statistical Software (v4.3.1; R Core Team 2021, R Foundation for Statistical Computing, Vienna, Austria) [35]. Visual inspection of boxplots was used to check datasets for the presence of outliers.

Data were analysed with a linear mixed model (LMM, lmerTest package, v3.1-3; [37] including group (level: HEMP and CON), period (level: period 1 and period 2), sequence (level: HEMP-CON or CON-HEMP) and block (levels: Block 1, Block 2, and Block 3) as fixed effects and the animal ID as random effect.

$${ϒ}_{ijkl}=\mu +\alpha_{i}+\beta_{j}+\gamma_{k}+\delta_{l}+\upsilon_{m}+\varepsilon_{ijkl},$$

where ϒ_ijkl_ is the dependent variable for *i*-th group, *j*-th period, the *k*-th sequence, and the *l*-th block; *μ* is the overall mean; *α*_i_ is the fixed effect of the *i*-th group; *β_j_* is the fixed effect of the *j*-th period; *γ_k_* is the fixed effect of the *k*-th sequence; *δ_l_* is the fixed effect of the *l*-th block; *ε_ijkl_* is the random error term; *υ_m_* is the random effect of the *m*-th animals.

For the statistical analysis of the antioxidant ability in feed extracts (2 groups with n = 3), a linear model including group and block as fixed effects was used.

$${ϒ}_{ij}=\mu +\alpha_{i}+\beta_{j}+\varepsilon_{ij},$$

where ϒ_ij_ is value of the dependent variable for the *i*-th group and the *j*-th block, *µ* is the mean, *α_i_* is the effect of the *i*-th group, *β_j_* is the effect of the *j*-th block, and *ε_ij_* is the residual.

For datasets containing multiple measurements (TEAC and FRAP of plasma) within a feeding period, the time point (day, 3 timepoints) and the interaction with the group (group × time point) were included as additional fixed effects.

$$\gamma_{ijklm}=\mu +\alpha_{i}+\beta_{j}+\gamma_{k}+\delta_{l}+\tau_{m}+\left(\alpha \tau \right)+\upsilon_{n}+\varepsilon_{ijklm}$$

where ϒ_ijklm_ is the dependent variable for *i*-th group, *j*-th period, the *k*-th sequence, *l*-th block and the *m*-th timepoint; *μ* is the overall mean; α_i_ is the fixed effect of the *i*-th group; *β_j_* is the fixed effect of the *j*-th period; *γ_k_* is the fixed effect of the *k*-th sequence; *δ_l_* is the fixed effect of the *l*-th block; *τ_m_* is the fixed effect of the *m*-th timepoint; (*ατ*) is the interaction between *i*-th group and *m*-th timepoint; *ε_ijklm_* is the random error term; *υ_n_* is the random effect of the *n*-th animals.

The residuals of the models were checked for normal distribution using the check_normality function and singularity using the check_singularity function of the performance package (v0.13.0 [38]). If the assumption of normality was violated, data were log2 transformed and re-evaluated. If singularity was detected, data were analysed using a linear model including group (level: HEMP and CON), period (level: period 1 and period 2), sequence (level: HEMP-CON or CON-HEMP) and the block (levels: Block 1, Block 2 and Block 3) as fixed effects. Thus, two treatments (HEMP and CON) in three replicates (Block 1 to 3) were evaluated.

Pairwise differences were tested by using the Tukey–Kramer test. For the fixed effect of interest, estimated marginal means (EMMs) and their standard errors (SE) were estimated. Effects and differences were considered significant at *p*  <  0.05 and considered as a trend at *p*  <  0.10.

To test the correlation between cannabinoid concentrations in milk and plasma, Kendall’s τ coefficient was computed, as samples were not normally distributed.

## 3. Results

### 3.1. Feed Constituents

To achieve isonitrogenous and isocaloric conditions between rations, the HEMP diet was formulated to contain 7.4% dried Santhica 27 leaves and 10% more rapeseed meal than the CON diet, whereas the CON diet contained 3.5% soybean meal and 1.8% straw (Supplementary Table S2). Leaves from the Santhica 27 variety contained less than 0.0005% Δ9-THC and 0.0003% THCA (Supplementary Table S3). Furthermore, hemp leaves had 6% and 30% more PUFA, 10% and 19% more SFA, and 15% and 49% less MUFA as compared to soybean meal and straw, respectively (Supplementary Table S4). However, the PUFA and SFA proportions were 24% and 10%, respectively, lower, but the MUFA proportion was 34% higher in rapeseed than in soybean meal. In addition, the total tannin and phenol contents were 2.6- and 4.6-fold greater in hemp and rapeseed than in soybean meal and straw, and the hemp flavonoid content was 8.5-fold greater than in straw and not detectable in both meals (Supplementary Table S5). However, the differences in the concentrations of condensed tannins, total tannins phenols, and flavonoids were smaller between the HEMP and CON TMR as compared to hemp leaves and soybean meal or straw (Supplementary Table S2).

### 3.2. Antioxidative Capacity of Feed Components and TMR

**Figure 1:**
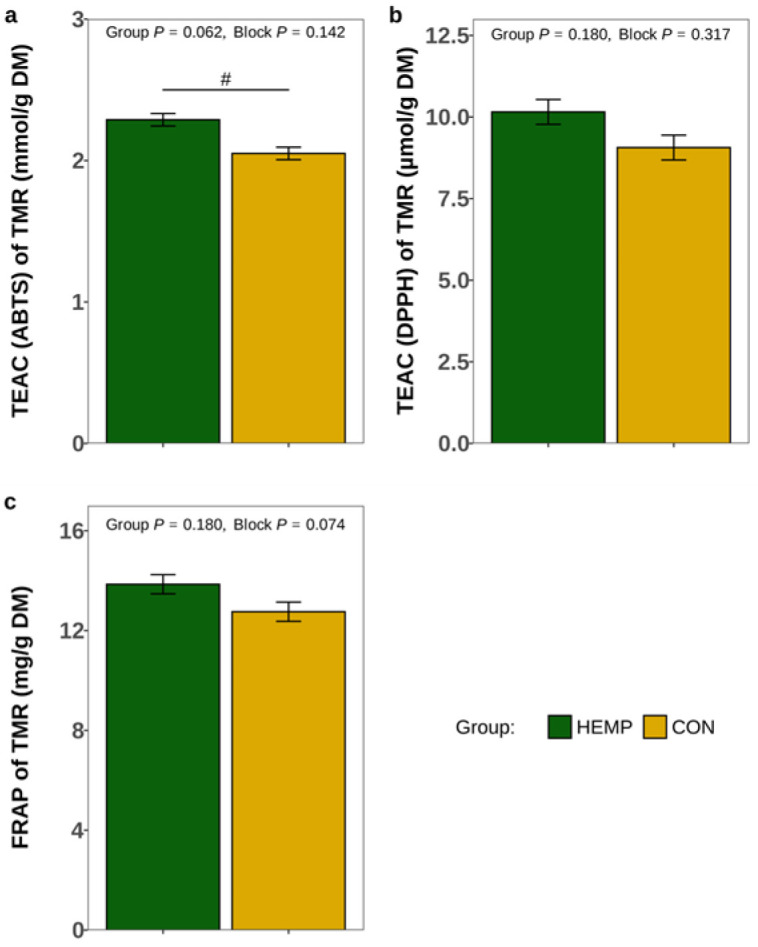
Trolox equivalent antioxidant capacity (TEAC) analysed by (**a**) 2,2′-azino-bis(3-ethylbenzothiazoline-6-sulfonic acid) (ABTS) assay, (**b**) 1,1-diphenyl-2-picrylhydrazil (DPPH) assay and (**c**) ferric-reducing antioxidant power (FRAP)) of the total mixed ration (TMR) containing either 7.4% Santhica 27 hemp leaves (HEMP, n = 3) or 3.5% soybean meal (CON, n = 3). # *p* < 0.1.

The TEAC and FRAP were greater in hemp leaves and rapeseed meal than in soybean meal and straw (Supplementary Table S5). Accordingly, the TEAC measured by the ABTS assay tended to be higher in the HEMP diet compared to the CON diet (*p* = 0.062, Figure 1a). However, TEAC measured by the DPPH and FRAP assays were not significantly different between the HEMP and CON diets (*p* > 0.1; Figure 1b,c).

### 3.3. Animal Performance

The inclusion of Santhica 27 hemp leaves in the diet reduced DMI by 1.7 kg/d (*p* = 0.011), milk yield by 1.1 kg/d (*p* < 0.044) and ECM by 1.4 kg/d (*p* = 0.042) (Table 1). However, the energy conversion efficiency expressed as ECM/ME intake was significantly higher in HEMP than in CON cows (*p* < 0.045). The analysis of milk constituents and milk somatic cell count, BW, mBW, BCS and BFT revealed no statistical differences between groups (*p* > 0.1). However, BW and mBW were affected by block (*p* < 0.05), and BCS tended to be affected by period (*p* = 0.097).

### 3.4. Plasma Cannabinoids and Antioxidative Capacity

Next, we examined if dairy cows absorb cannabinoids from Santhica hemp leaves and if the post-absorptive metabolism can make use of their greater antioxidative capacity. After feeding the HEMP diet for 14 days, the highest plasma cannabinoid concentration was found for CBGA, amounting to 181 ng/mL, followed by CBDA with a concentration of 5.861 ng/mL (Table 2). The plasma THC concentration of the HEMP group was 0.003 ng/mL and, thus, the lowest cannabinoid concentration. Surprisingly, in three cows of the CON group, CBDA and CBGA were detected in mean concentrations of 0.003 ng/mL and 0.038 ng/mL, respectively. In one further CON animal, a THC concentration of 0.01 ng/mL was detected.

**Figure 2:**
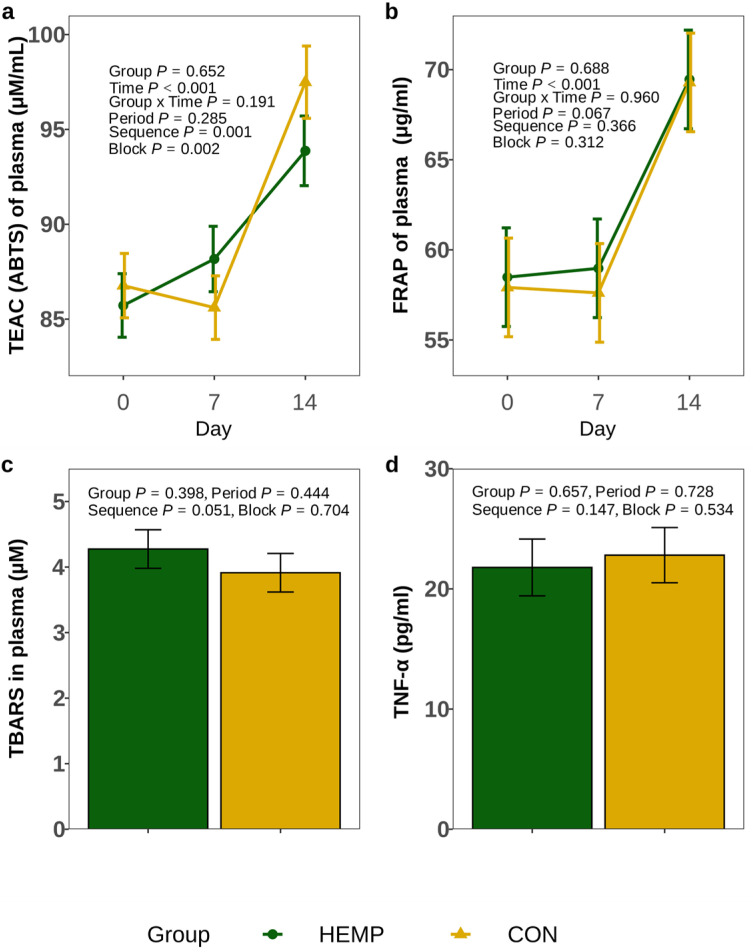
Trolox equivalent antioxidant capacity (TEAC) analysed by (**a**) a 2,2′-azino-bis(3-ethylbenzothiazoline-6-sulfonic acid) (ABTS) assay*, (**b**) the ferric-reducing antioxidant power (FRAP), (**c**) the thiobarbituric acid reactive substances (TBARS) and (**d**) the tumour necrosis factor alpha (TNF-α) concentrations in plasma obtained on day 14 from cows fed a diet containing 7.4% hemp leaves (HEMP, n = 12) or 3.5% soybean meal (CON, n = 12). n = 12 for each group for TEAC, FRAP and TBARS; n = 11 CON and n = 10 HEMP cows for TNF-α. * Data were log-transformed. Back-transformed data are shown for interpretation.

In addition, the plasma TEAC analysed by the ABTS assay and the FRAP did not differ between HEMP and CON groups (*p* > 0.1, Figure 2a,b). In addition, plasma TBARS concentrations were not different between cow groups (*p* = 0.398, Figure 2c).

### 3.5. Plasma Amino Acid Concentrations

To assess if HEMP feeding affects excitotoxic amino acid concentration and the amino acid-mediated antioxidative capacity [39], we next analysed the plasma-free amino acid profile. Cows fed the HEMP diet had 9.6% lower (*p* < 0.002) plasma π-methylhistidine and α-aminoadipic acid and tended to have a 12.3% lower isoleucine plasma concentration compared to the CON group (*p* = 0·056, Table 3). On the other hand, HEMP feeding increased plasma anserine concentration by 41.3% (*p* < 0.001). The period affected (*p* < 0.05) plasma aspartic acid, π-methylhistidine, and anserine concentrations and tended (*p* = 0.071) to affect arginine. In addition, plasma π-methylhistidine concentrations were (*p* < 0.05), and aspartic acid tended (*p* = 0.065) to be affected by the feeding sequence. Furthermore, the block affected plasma aspartic acid, τ-methylhistidine, alanine, taurine, and proline concentrations (*p* < 0.050), while histidine tended to be affected by the block (*p* = 0.064). The complete table of all measured plasma-free amino acid concentrations can be found in Supplementary Table S6.

### 3.6. Inflammatory Parameters

The analysis of white blood cells obtained on day 14 revealed that feeding the HEMP diet resulted in a 1.3-fold higher mRNA abundance of the p65 coding RELA proto-oncogene, which is involved in NF-κB subunit heterodimerisation (*p* = 0.014, Table 4). Despite this, the TNFA mRNA abundance tended to be lower in HEMP than in CON cows (*p* = 0.078), whereas the plasma TNF-α concentrations did not significantly differ between groups (*p* = 0.657, Figure 2d). In addition, the transcript levels of interleukin-1beta (IL1B) and toll-like receptor 4 (TLR4) were also not different between groups (*p* > 0.1; Table 4).

### 3.7. Milk Cannabinoid Concentrations

The cannabinoid concentrations in pooled milk samples taken from the evening milking on d 11 and morning milking on d 12 are presented in Table 2. None of the cannabinoids analysed were detected in the milk of the CON group. The highest milk cannabinoid concentrations were observed for CBGA (1.55 ng/mL) and CBD (1.23 ng/mL), whereas the THCA concentration was lowest (0.01 ng/mL) in HEMP cows. No correlations were observed between plasma and milk cannabinoid concentrations of HEMP cows, with the exception of CBD, where a trend towards a moderate correlation was found (τ = 0.443, *p* = 0.054, Table 5).

### 3.8. Milk Fatty Acid Profile

Feeding the HEMP compared to the CON diet increased the proportion of milk heptadecanoic acid (C17:0) by 10.0% (*p* = 0.006), docosanoic acid (*C*22:0) by 18.5% (*p* = 0.040) and tended to increase arachidic acid (C20:0) by 6.4% (*p* = 0.064, Table 6). However, the sum of saturated fatty acids (SFA) did not differ between groups (*p* = 0.838, Table 7). Cows of the HEMP group produced a 15.9% higher proportion of the monounsaturated fatty acid (MUFA) *cis*-vaccenic acid (*C*18:1*cis*-11) and a 13.1% higher proportion of 11-eicosenoic acid (C20:1*cis*-11) compared to the CON group (*p* < 0.050, Table 6). Yet, the sum of MUFA was comparable between groups (*p* = 0.780, Table 7).

The PUFA α-linolenic acid (*C*18:3n-3) and arachidonic acid (C20:4n-6) proportions were 29.8% and 7.8%, respectively, higher in the HEMP compared to the CON group (*p* < 0.01, Table 6). In addition, the milk proportion of n-6-docosapentaenoic acid (*C*22:5n-6) tended to be 25% higher in HEMP than in CON cows (*p* = 0.071, Table 6). In contrast, the conjugated linoleic acid (*C*18:2*cis*-9.trans-11) proportion in milk was 11.8% lower in the HEMP group (*p* = 0.004). The sum of n-3 PUFA was 23.0% higher (*p* < 0.001) and the sum of PUFA tended to be higher (*p* = 0.071, Table 7) in the milk of HEMP compared to CON cows. Moreover, the n-6/n-3 ratio and the LA/ALA ratio were lower in HEMP compared to CON cows (*p* < 0.001), while the PUFA/SFA ratio tended to be higher in the HEMP group (*p* = 0.053, Table 7). However, the AI, TI, HPI and h/H indices, as well as the proportions of trans-fatty acids (TFA), did not differ between groups (*p* > 0.1, Table 7). The period affected *C*18:1*trans*-11, *C*22:2n-6, *C*22:4n-6 and TFA proportions in whey (*p* < 0.05). In addition, *C*16:1*cis*-9, *C*18:2n-6, *C*18:3n-3 and *C*18:4n-3 were affected by feeding sequence (*p* < 0.05), and C15:0, C13:0, *C*18:2*cis*-9.trans-11, *C*22:2n-6, *C*22:4n-6 and n-6/n-3 tended to be affected by sequence (*p* < 0.100). The complete table of all measured milk fatty acids and amino acids can be found in Supplementary Table S7.

### 3.9. Whey FRAP and Amino Acid Concentrations

**Figure 3:**
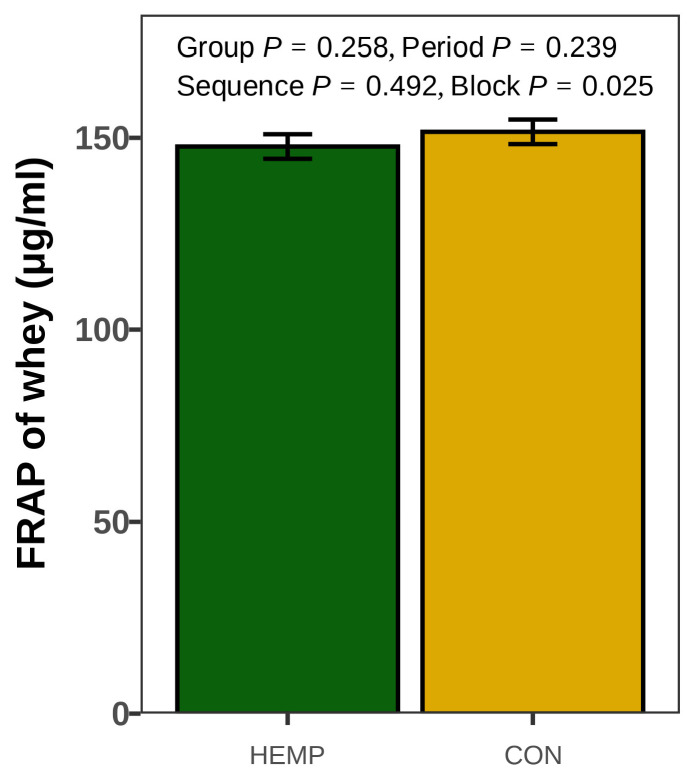
The ferric-reducing antioxidant power (FRAP) of whey in samples pooled from the evening milking on d 11 and morning milking on d 12 from cows fed a diet containing 7.4% hemp leaves (HEMP, n = 12) or 3.5% soybean meal (CON, n = 12).

The ferric-reducing ability of whey was comparable in both groups (*p* > 0.100); however, the block affected the FRAP of whey (*p* = 0.025; Figure 3).

The sum of amino acid concentrations in whey protein did not differ between the groups (*p* > 0.100, Table 8). Yet, feeding a diet containing hemp leaves increased γ-aminobutyric acid (GABA) concentration in whey protein by 9.4% (*p* = 0.013, Table 8) and tended to decrease α-aminobutyric acid concentration by 20.9% (*p* = 0.055). The ß-alanine and GABA concentrations in whey tended to be affected (*p* < 0.100), and asparagine was affected by period (*p* < 0.006, Table 4). The sequence affected the whey concentrations of histidine (*p* < 0.050) and tended to affect the concentrations of arginine, glutamine, glutamic acid, leucine, hydroxy proline, and the total sum of amino acid concentrations (*p* < 0.100). Furthermore, there was an effect of the block on whey cysteine, α-aminoadipic acid, asparagine, glycine, phenylalanine, lysine, hydroxy proline, and proline concentrations (*p* < 0.050) and a trend for τ-methylhistidine and methionine concentrations (*p* < 0.100). The complete list of all measured whey amino acids can be found in the Supplementary Table S8.

## 4. Discussion

### 4.1. Diet Composition and Feed Intake

Feeding 7.4% industrial hemp leaves of the variety Santhica 27 reduced the DMI of HEMP cows. Because Santhica 27 hemp leaves contained negligible amounts of THC and CBN, it appears that the DMI of cows is not suppressed by these two cannabinoids. This conclusion appears to be in contrast to the food intake stimulating effect of THC and CBN administered in a single dose to rodents; however, the stimulatory action lasted only for a few hours [40,41]. Thus, cannabinoids other than THC and CBN must account for the reduction in DMI in HEMP cows. Repetitive weekly administration of CBD, but THC and CBN has also been shown to reduce the food consumption of rats up to 4 days after dosing [42]. The latter study suggests that CBD, which was contained in Santhica 27 leaves in appreciable quantities of >50 mg/kg and detectable in plasma, elicited hypophagia in HEMP cows.

Besides CBD, industrial hemp silage [6] contains substantial amounts of CBN and THC, which also likely contributed to the reduction in feed intake. Thus, the total cannabinoid concentration rather than individual cannabinoic substances seems to suppress the DMI of cows, and this assumption is supported by a study in lambs showing that the inclusion of 20% but not 10% SHB in the diet reduced feed intake over a period of 4 weeks [17].

A further reason for the reduction in DMI could be the modulating effect of cannabinoids on smell and taste. Various plant cannabinoids in rats [42], as well as the endocannabinoid N-arachidonoylethanolamide in lactating cows [43], bind to the cannabinoid receptor 1 (CB1) and modulate the olfactory perception by amplifying the preference for sweet taste. Possibly, the amounts of starch and free sugars in the HEMP diet were not sufficient to meet the demand for a sweet taste after hemp ingestion. If the addition of sweeteners to a hemp-containing diet would prevent the decline in cow’s feed intake, it still needs to be explored.

Further, Cannabis sativa leaves contain a bunch of terpenes and terpenoids, of which some, for example, myrcene, possess a unique smell or narcotic-like properties [44]. Although their actions in cows have not been studied, we cannot exclude an effect of terpenes or terpenoids on feed palatability or tiredness, which would promote the reduction in DMI. Irawan et al. (2024) have already mentioned that hemp in the form of SHB impairs palatability for cows [7].

Additionally, condensed tannins were reported to diminish the feed intake of ruminants when the dietary concentration exceeds 50 g/kg of DM [45]. The concentrations of condensed tannins of the CON and HEMP diets did not exceed this threshold. Thus, condensed tannins can be excluded as a reason for the lower DMI of HEMP cows.

### 4.2. Milk Constituents

The lower DMI and, therefore, ME intake resulted in a lower milk and ECM yield of the HEMP group. Similarly, Wagner et al. (2022) [6] reported a decline in feed intake and an accompanied reduction in milk yield when adding a cannabinoid-rich hemp silage to the diet. In contrast, Irawan et al. (2024) [7] found no effect on milk yield despite a reduction in feed intake on a diet containing 13% SHB, but the ECM/DMI ratio was numerically higher in SHB-supplemented cows. In our study, the HEMP group exhibited a greater energy conversion ratio (ECM/ME intake) than CON cows, presumably because hemp did not serve as a basis for the development of the ME estimation equation and was underestimated.

Despite the lower DMI, feeding the HEMP diet did not affect milk fat, protein or lactose concentrations but altered the milk fatty acid composition. Specifically, for the proportion of milk long-chain fatty acids (*C*18:3n-3, *C*18:1*cis*-11, C20:0; C20:1*cis*-11, C20:4n-6, *C*22:0), the total n-3 PUFA and the PUFA/SFA ratio was or tended to be higher, whereas for the proportion of CLA, the n-6/n-3 PUFA ratio and the LA/ALA ratio were lower in HEMP than CON cows. These changes were primarily due to the different percentages of total SFA, MUFA, n-6 and n-3 PUFA, as well as the n-6/n-3 ratio in Santhica 27 hemp leaves in comparison to soybean meal and straw present in the CON ration. It has been shown that the supplementation of n-3 PUFA facilitates the portion of preformed long-chain fatty acids (*C*18:3n-3, C20:0) and reduces the milk n-6/n-3 PUFA ratio [46], and this finding corresponds to the present results. Feeding HEMP increased the C17:0 proportion compared to CON. The C17:0 fatty acid is mainly synthesised by elongation of ruminal-derived propionate or n-valerate [47]. However, ruminal propionate and n-valerate concentrations were only numerically higher in HEMP and CON cows [8], and thus, a small proportion could also be produced by the mammary gland [48].

The *C*12:0, *C*14:0 and *C*16:0 milk fatty acids were not reduced after HEMP feeding, and this is the reason for the absence of differences in HPI and AI between HEMP and CON groups. Further, the difference in the mean sum of milk n-3 PUFA proportions and the mean sum of total PUFA proportions between HEMP and CON cows was not large enough to secure differences in the nutritional h/H and TI. However, Irawan et al. (2024) stated that SHB-fed cows reveal a tendency for a higher h/H and a lower TI, but this effect was presumably due to the higher n-6/n-3 ratio (6.4) of SHB and the greater portion of hemp biomass (13%) than used in the present study [7]. The higher proportion of n-3 PUFAs and the lower n-6/n-3 PUFA ratio in the milk of HEMP cows may be beneficial for human nutrition, as n-3 PUFA exert antioxidant and anti-inflammatory properties and have a positive impact on the cardiovascular system [49,50].

The LA/ALA ratio was found to be significantly lower in HEMP (3.7:1) than in CON (4.6:1) cows. A low LA/ALA ratio is favourable in human nutrition, as a low LA/ALA ratio is associated with anti-inflammatory effects and the prevention of cardiovascular diseases, although a target LA/ALA ratio is still under debate [51,52,53]. However, the current nutritional recommendations for the LA/ALA ratio for infants range between 5:1 and 15:1 [54], and neither the milk from HEMP nor CON cows meet these recommendations. Furthermore, we found numerous cannabinoids, particularly CBGA, CBD, and CBC, with the highest concentrations, but THCA, Δ^9^-THC and CBDA had the lowest concentrations in milk. After feeding daily 0.84 to 1.68 kg of DM industrial hemp silage to dairy cows for 6 days, CBD and Δ^9^-THC concentrations in milk reached levels up to 1000 µg/kg and 400 µg/kg, respectively [6]. The milk CBD level is comparable to our result, showing that HEMP cows consumed on average 1.39 kg of DM Santhica 27 leaves per day to excrete 1231 µg CBD per litre milk, whereas the milk Δ^9^-THC concentration (0.075 ng/mL) was 530-fold lower than in the [6] study. The very low milk Δ^9^-THC concentration is due to the very low Δ^9^-THC content of Santhica 27 leaves. As the milk of HEMP cows contained 0.075 μg/L THC, an infant (5 kg BW) would have to consume 68 kg and an adult (60 kg BW) 822 kg of milk to exceed the acute reference dose. Despite the high CBD, CBC and CBGA concentrations in the milk of HEMP cows, the FRAP of whey was not higher than in CON cows, likely because isoflavones, such as genistein, equol, enterolactone, formononetin, and daidzein [55] ingested with soybean meal by CON cows were also transferred into milk to exert a similar antioxidant power.

Whey from HEMP cows contained higher GABA concentration than milk from CON cows. GABA serves as the main inhibitory neurotransmitter to reduce neuronal excitability. Oral uptake of 600 mg CBD increased the GABA concentrations in the brains of healthy adult humans [56]. It seems possible that the daily uptake of 73 mg CBD by HEMP cows increased the neuronal GABA concentrations, which might explain their longer resting times [8]. Prolongation of resting times upon industrial hemp feeding has also been reported by Kleinhenz et al. (2022) [57]. However, we did not observe increased plasma GABA concentrations in HEMP cows, either because the GABA analysis was confounded by the coincidence of iso-aminobutyric acid, that GABAergic neurons of the mammary gland are activated, or that the mammary gland serves as a sink for circulating GABA.

Furthermore, we found different whey but not plasma α-aminobutyric acid concentrations between groups. Recent studies showed the involvement of α-aminobutyric acid in glutathione metabolism, but it can also be produced from methionine, threonine, serine, glycine, and branched-chain amino acids [58]. Hence, the tendency for lower α-aminobutyric acid concentrations in the whey of HEMP cows might reflect dysregulation of these metabolic pathways in the mammary gland or other excretion organs.

### 4.3. Plasma Antioxidative Capacity, Inflammatory Parameters and Amino Acids

Although the HEMP diet possessed an on average 12% higher TEAC concentration (as measured by the ABTS assay), the 8% lower DMI resulted in a similar amount of ingested TEAC as in the CON group. Accordingly, HEMP cows could not take advantage of TEAC ingested with Santhica 27 leaves, as seen by the plasma TEAC, FRAP and TBARS concentrations, which did not differ between the HEMP and CON groups. Similarly, plasma FRAP, reactive oxygen metabolites and thiol group concentrations [7] or plasma TEAC [14] of cows fed SHB were not different from control groups despite an up to 1.75-fold higher hemp portion in the ration than in our study. It seems unlikely that the enriched cannabinoid concentrations in the plasma of HEMP cows exerted any major antioxidative or anti-inflammatory effect; otherwise, one would expect greater TEAC or FRAP values and lower TBARS and TNF-α concentrations in these animals. Furthermore, the *TNFA* mRNA abundance would have been significantly lower in HEMP cows, but only a trend was found.

By contrast, the *RELA* mRNA expression was significantly higher in the HEMP group, suggesting that Santhica 27 feeding increases the inflammatory response. Our results seem to contrast the often proven antioxidative and anti-inflammatory effects of cannabinoids in rodents and humans [59] and the decrease in plasma IL-1β concentration of Holstein cows fed SHB [14]. However, the cows investigated in the present study had no clinical signs of acute inflammation, which could have been reduced through plasma cannabinoid concentrations. Moreover, the CON diet contained soybean meal, which in turn is a source of isoflavones exhibiting potent antioxidative properties. Supplementing the diet with 200 to 400 mg/day of the isoflavone daidzein reduced the total antioxidative capacity and TBARS plasma concentrations in heat-stressed cows [60]. Thus, the antioxidative capacity caused by plasma cannabinoids in the HEMP group could have been balanced by the antioxidative capacity induced by soybean-derived isoflavones. Similarly, Wang et al. (2023) found no effect on the plasma oxidative status when Holstein cows were fed a TMR either containing SHB or alfalfa hay [14], the latter including various compounds with antioxidant activity [61].

Feeding hemp leaves increased the plasma anserine concentration. Anserine is formed by methylation of carnosine catalysed by carnosine *N*-methyltransferase [62,63]. However, the plasma carnosine concentrations were comparable between the HEMP and CON groups, suggesting that the carnosine *N*-methyltransferase activity is not affected by the HEMP diet. On the other hand, anserine is degraded by carnosinases (CN1 and CN2) to yield π-methylhistidine and β-alanine [64,65]. Thus, the increased plasma anserine concentration could be due to diminished CN1 and CN2 activities. This assumption is supported by the fact that the plasma π-methylhistidine concentration tended to be lower in HEMP compared to CON cows. However, it is currently unknown whether cannabinoids or other hemp constituents reduce the enzyme activity of carnosinases or if the lower plasma π-methylhistidine reflects diminished skeletal muscle catabolism. The higher plasma anserine concentrations of HEMP cows could also be due to changes in the rumen microbiome. In yaks, metabolomics analyses have shown a negative correlation between the relative ruminal abundance of the genus *Fibrobacter* and anserine levels in rumen fluid [66]. Although the cause–effect relationship between ruminal and plasma anserine concentration appears questionable, the analysis of the rumen microbiota from HEMP and CON cows deserves further investigation.

Carnosine and anserine are antioxidants that act as reducing agents and free radical scavengers, thereby preventing lipid peroxidation [67]. However, the higher plasma anserine concentration of HEMP cows was not accompanied by a higher TEAC of plasma, suggesting again that various antioxidant compounds in the plasma of CON cows contribute to a comparable TEAC level between HEMP and CON cows.

Besides anserine, seven amino acids, including tryptophan, methionine, histidine, lysine, cysteine, arginine and tyrosine, revealed a greater antioxidative capacity than the other 13 proteinogenic amino acids [39]. However, in our study, none of these amino acid concentrations differed between HEMP and CON cows. Instead, the plasma α-aminoadipic acid concentration was lower in HEMP than in the CON cows. α-Aminoadipate, the salt prevalent at physiological pH, is involved in lysine degradation, producing glutamate as a side-product. However, neither plasma lysine nor glutamate concentrations differ between the groups. The lack of differences in plasma glutamate and aspartate concentrations between groups indicates that HEMP feeding did not affect excitatory amino acid responses, at least not in plasma. Feeding hemp also resulted in a trend towards lower plasma isoleucine concentrations. Isoleucine is implicated in milk protein synthesis [68]; however, no differences in milk protein or whey isoleucine concentrations were observed in our study. Thus, the reason for the tending lower plasma isoleucine concentrations in HEMP cows remains to be clarified in future studies.

### 4.4. Limitations

In the present study, significant period, sequence and block effects were observed for various parameters. These effects may be attributable to environmental influences, like temperature and season, which may have an effect on feed intake. In addition, variations in the concentration of nutrients and secondary plant substances in the diet over the course of the experiment may have had an influence on the measured parameters and thus led to significant block, period or sequence effects. Last but not least, the cows examined in this study were in mid-lactation and clinically healthy, so they did not show increased oxidative stress and inflammation levels, which meant that the full reduction potential for these parameters after supplementation with hemp leaves could not be determined.

## 5. Conclusions

To summarise and conclude, the inclusion of hemp leaves with very low tetrahydrocannabinol (THC) content into the diet reduced dry matter intake and, thus, milk performance. On the molecular level, the inclusion of hemp leaves reduced the milk n-6/n-3 polyunsaturated fatty acids (PUFA) ratio and, as a trend, the tumour necrosis factor mRNA expression of white blood cells. On the other hand, feeding Santhica 27 hemp leaves increased the energy conversion efficiency, non-THC cannabinoid concentrations in plasma and milk, various parameters characterising the plasma antioxidative status, the RELA Proto-Oncogene, NF-KB Subunit mRNA expression of white blood cells, and the proportion of milk n-3 PUFA as compared to the inclusion of soybean meal. These results indicate that Santhica 27 hemp feeding increased the antioxidative capacity of dairy cows, which potentially helps reduce oxidative damage in phases of increased oxidative stress. In addition, although supplementation of 7.4% Santhica 27 hemp leaves to the diet of lactating dairy cows resulted in a milk THC concentration of 0.075 μg/L, this concentration is unlikely to exceed the acute reference dose for humans.

## Figures and Tables

**Table 1:** Body constitution, performance and milk composition of cows fed a diet containing 7.4% hemp leaves (HEMP, n = 12) or 3.5% soybean meal (CON, n = 12).

	EMMs ± SE		*p*-Value			
Item	HEMP	CON	Group	Period	Sequence	Block
**Body constitution**						
BW, kg	662 ± 14.2	669 ± 14.2	0.142	0.105	0.464	0.032
mBW, kg	130 ± 2.07	130 ± 2.07	0.135	0.107	0.472	0.032
BCS	3.4 ± 0.19	3.4 ± 0.19	0.843	0.097	0.765	0.241
BFT, mm	0.91 ± 0.11	0.94 ± 0.11	0.133	0.373	0.895	0.704
**Performance**						
DMI, kg	18.7 ± 0.43	20.4 ± 0.43	0.011	0.163	0.600	0.217
Milk yield, kg	28.6 ± 1.23	29.7 ± 1.23	0.044	0.129	0.628	0.318
ECM, kg	27.4 ± 1.03	28.8 ± 1.03	0.042	0.906	0.628	0.280
ECM/ME intake	0.129 ± 0.003	0.124 ± 0.003	0.045	0.032	0.457	0.081
**Milk composition**						
Fat, %	3.57 ± 0.13	3.66 ± 0.13	0.484	0.111	0.999	0.816
Protein, %	3.57 ± 0.06	3.59 ± 0.06	0.681	0.177	0.224	0.608
Urea, mg/L	172 ± 7.29	180 ± 7.29	0.407	0.161	0.691	0.267
Lactose, %	4.94 ± 0.03	4.96 ± 0.03	0.242	0.703	0.979	0.243
Somatic cell count *, × 1000/mL	72 ± 16.2	80 ± 17.1	0.870	0.217	0.038	0.404

EMMs: estimated marginal means; SE: standard error; BW, body weight; mBW, metabolic body weight; BFT, back fat thickness; DMI: dry matter intake; ECM: energy corrected milk; ECM/ME: energy corrected milk/metabolisable energy. * Data were log-transformed. Back-transformed data are shown for interpretation.

**Table 2:** Plasma ^1^ and milk ^2^ cannabinoid concentration of cows fed a diet containing 7.4% Santhica 27 hemp leaves (HEMP, n = 12) or 3.5% soybean meal (CON, n = 12) (mean ± SD).

	Plasma		Milk	
Cannabinoid, µg/L	HEMP	CON	HEMP	CON
CBC	0.133 ± 0.093	0.000 ± 0.000	0.922 ± 0.910	0.000 ± 0.000
CBD	0.488 ± 0.324	0.000 ± 0.000	1.231 ± 0.295	0.000 ± 0.000
CBDA	5.861 ± 1.602	0.003 ± 0.007	0.053 ± 0.018	0.000 ± 0.000
CBG	1.603 ± 1.069	0.000 ± 0.000	0.273 ± 0.077	0.000 ± 0.000
CBGA	181.48 ± 41.13	0.038 ± 0.096	1.546 ± 0.480	0.000 ± 0.000
Δ9-THC	0.003 ± 0.007	0.001 ± 0.003	0.075 ± 0.023	0.000 ± 0.000
THCA	0.213 ± 0.141	0.000 ± 0.000	0.008 ± 0.006	0.000 ± 0.000

CBC: cannabichromene; CBD: cannabidiol; CBDA: cannabidiolic acid; CBG: cannabigerol; CBGA: cannabigerolic acid; Δ9-THC: tetrahydrocannabinol; THCA: tetrahydrocannabinolic acid; 1: samples taken after 14 days of each feeding period; 2: pooled samples taken from the evening and morning milking on days 11 and 12 of each feeding period.

**Table 3:** Selected plasma-free amino acid concentrations of cows fed a diet containing 7.4% Santhica 27 hemp leaves (HEMP, n = 12) or 3.5% soybean meal (CON, n = 12) on d 14 of the feeding period.

	EMMs ± SE		*p*-Value			
Amino Acid, µM	HEMP	CON	Group	Period	Sequence	Block
Aspartic acid	5.5 ± 0.25	5.4 ± 0.25	0.721	0.023	0.065	0.010
α-Aminoadipic acid	4.8 ± 0.34	5.5 ± 0.34	0.039	0.298	0.444	0.367
Histidine	47.1 ± 5.15	46.0 ± 5.15	0.775	0.304	0.658	0.064
Arginine	82.3 ± 6.27	82.8 ± 6.27	0.943	0.071	0.646	0.305
π-Methylhistidine	3.9 ± 0.27	4.4 ± 0.27	0.002	0.042	0.029	0.550
τ-Methylhistidine	5.4 ± 0.19	5.4 ± 0.19	0.724	0.213	0.838	0.016
Alanine *	251 ± 10.0	259 ± 10.0	0.445	0.279	0.235	0.019
Taurine	41.240 ± 2.52	47.3 ± 2.52	0.107	0.250	0.800	0.017
Anserine	0.8 ± 0.05	0.6 ± 0.05	<0.001	0.036	0.804	0.874
α-aminobutyric acid	16.8 ± 1.1	17.8 ± 1.1	0.515	0.904	0.063	0.767
Isoleucine	107 ± 8.3	122 ± 8.3	0.056	0.238	0.622	0.404
Proline	130.0 ± 3.5	133.0 ± 3.5	0.488	0.180	0.937	<0.001

* Data were log-transformed. Back-transformed data are shown for interpretation. EMMs, estimated marginal means; SE, standard error.

**Table 4:** Relative mRNA abundance of genes related to immunologic function in buffy coat cells extracted on day 14 from cows fed a diet containing 7.4% Santhica 27 hemp leaves (HEMP, n = 6) or 3.5% soybean meal (CON, n = 6).

	EMMs ± SE		*p*-Values			
Gene	HEMP	CON	Group	Period	Sequence	Block
*TNF*	0.68 ± 0.117	1.06 ± 0.113	0.078	0.162	0.188	0.642
*RELA*	1.23 ± 0.059	0.99 ± 0.055	0.014	0.007	0.013	0.704
*IL1B*	1.01 ± 0.228	1.24 ± 0.218	0.296	0.359	0.532	0.426
*TLR4*	0.77 ± 0.094	0.87 ± 0.872	0.341	0.187	0.972	0.205

EMMs, estimated marginal means; SE, standard error; TNF, tumour necrosis factor; RELA, RELA proto-oncogene, NF-KB subunit; IL1B, interleukin-1beta; TLR4, toll-like receptor 4.

**Table 5:** Kendall’s correlations between milk and plasma cannabinoid concentration of cows fed a diet containing 7.4% Santhica 27 hemp leaves (HEMP, n = 12) or 3.5% soybean meal (CON, n = 12).

	Kendall’s τ Coefficient	*p*-Value
CBC	0.326	0.167
CBD	0.443	0.054
CBDA	−0.273	0.278
CBG	0.123	0.630
CBGA	−0.121	0.631
THC	0.404	0.139
THCA	0.064	0.867

CBC: cannabichromene; CBD: cannabidiol; CBDA: cannabidiolic acid; CBG: cannabigerol; CBGA: cannabigerolic acid; THC: tetrahydrocannabinol; THCA: tetrahydrocannabinolic acid.

**Table 6:** Selected saturated fatty acids (SFA), monounsaturated fatty acids (MUFA) and polyunsaturated fatty acids (PUFA) (mg/100 g) of cows fed a diet containing 7.4% Santhica 27 hemp leaves (HEMP, n = 12) or 3.5% soybean meal (CON, n = 12).

	EMMs ± SE		*p*-Value			
	HEMP	CON	Group	Period	Sequence	Block
**Saturated fatty acids** **(SFA)**						
*C*14:0	12.70 ± 0.274	12.80 ± 0.274	0.683	0.953	0.481	0.954
*C*16:0	31.00 ± 0.803	31.50 ± 0.803	0.291	0.024	0.527	0.611
C17:0	0.58 ± 0.020	0.53 ± 0.020	0.006	0.789	0.805	0.542
*C*18:0	9.17 ± 0.451	9.26 ± 0.451	0.825	0.134	0.461	0.571
C20:0	0.18 ± 0.007	0.17 ± 0.007	0.070	0.113	0.794	0.363
C21:0	0.06 ± 0.004	0.06 ± 0.004	0.379	0.788	0.252	0.273
*C*22:0	0.06 ± 0.004	0.05 ± 0.004	0.040	0.450	0.952	0.307
**Monounsaturated fatty acids (MUFA)**						
*C*18:1*trans*-11	1.14 ± 0.075	1.16 ± 0.075	0.863	0.035	0.885	0.692
*C*18:1*cis-*9	22.80 ± 0.709	22.30 ± 0.709	0.440	0.988	0.625	0.851
*C*18:1*cis*-11	1.48 ± 0.053	1.26 ± 0.053	0.011	0.495	0.646	0.525
C20:1*cis*-11	0.14 ± 0.004	0.12 ± 0.004	0.004	1.000	0.784	0.942
**Polyunsaturated fatty acids (PUFA)**						
*C*18:2n-6 (LA)	2.17 ± 0.058	2.08 ± 0.058	0.239	0.210	0.015	0.394
*C*18:3n-3 (LNA)	0.59 ± 0.015	0.46 ± 0.015	<0.001	0.428	0.045	0.178
*C*18:2*cis*-9.trans-11 (CLA)	0.54 ± 0.016	0.61 ± 0.016	0.004	0.880	0.096	0.001
C20:4n-6	0.13 ± 0.004	0.12 ± 0.004	0.001	0.624	0.242	0.164
*C*22:2n-6	0.04 ± 0.006	0.04 ± 0.006	0.894	0.034	0.097	0.648
C20:5n-3(EPA)	0.04 ± 0.003	0.04 ± 0.003	0.150	0.828	0.865	0.350
*C*22:5n-6	0.12 ± 0.017	0.09 ± 0.017	0.071	0.408	0.123	0.152

EMMs, estimated marginal means; SE, standard error.

**Table 7:** The sum of SFA, MUFA, PUFA (mg/100 g) and milk health indices of cows fed a diet containing 7.4% Santhica 27 hemp leaves (HEMP, n = 12) or 3.5% soybean meal (CON, n = 12).

	EMMs ± SE		*p*-Value			
	HEMP	CON	Group	Period	Sequence	Block
Sum SFA	66.30 ± 0.838	67.10 ± 0.838	0.276	0.860	0.862	0.905
Sum MUFA	29.70 ± 0.780	29.00 ± 0.780	0.398	0.942	0.937	0.892
Sum PUFA	4.08 ± 0.100	3.88 ± 0.100	0.071	0.468	0.373	0.581
Sum n-3 PUFA	0.85 ± 0.030	0.69 ± 0.030	<0.001	0.206	0.651	0.659
Sum n-6 PUFA	2.70 ± 0.078	2.58 ± 0.078	0.214	0.587	0.267	0.277
Milk fat, %	2.80 ± 0.104	2.90 ± 0.104	0.416	0.495	0.772	0.896
n-6/n-3	3.22 ± 0.092	3.78 ± 0.092	<0.001	0.318	0.072	0.181
AI	2.61 ± 0.105	2.71 ± 0.105	0.290	0.663	0.937	0.912
TI	0.35 ± 0.013	0.36 ± 0.013	0.303	0.703	0.975	0.931
h/H	0.67 ± 0.031	0.64 ± 0.031	0.213	0.329	0.578	0.853
PUFA/SFA	0.062 ± 0.002	0.058 ± 0.002	0.053	0.486	0.470	0.716
HPI	0.39 ± 0.015	0.37 ± 0.015	0.266	0.635	0.918	0.886
LA/ALA	3.67 ± 0.087	4.60 ± 0.087	<0.001	0.179	0.740	<0.001
TFA	1.46 ± 0.079	1.47 ± 0.079	0.864	0.035	0.892	0.780

EMMs, estimated marginal means; SE, standard error; SFA: saturated fatty acids; MUFA: monounsaturated fatty acids; PUFA: polyunsaturated fatty acids. AI: Atherogenic index: [*C*12:0 + (4 × *C*14:0) + *C*16:0]/ΣUFA. TI: Thrombogenic index: (*C*14:0 + *C*16:0 + *C*18:0)/[(0.5 × ΣMUFA) + (0.5 × Σn-6 PUFA) + (3 × Σn-3 PUFA) + (n-3/n-6)]. h/H: Hypocholesterolemic/hypercholesterolemic ratio (*cis*-*C*18:1 + ΣPUFA)/(*C*12:0 + *C*14:0 + *C*16:0). PUFA/SFA: Polyunsaturated fatty acid/saturated fatty acid ratio. HPI: Health-promoting index ΣUFA/[*C*12:0 + (4 × *C*14:0) + *C*16:0]. LA/ALA: Linoleic acid/α-linolenic acid ratio *C*18:2 n-6/*C*18:3 n-3. TFA: Trans fatty acid.

**Table 8:** Selected whey-free amino acid concentrations of cows fed a diet containing 7.4% Santhica 27 hemp leaves (HEMP, n = 12) or 3.5% soybean meal (CON, n = 12) in pooled milk samples collected on d 11 and 12 of the feeding period.

	EMMs ± SE		*p*-Value			
Amino Acid, µmol/L	HEMP	CON	Group	Period	Sequence	Block
Glutamic acid	543 ± 46.2	554 ± 46.2	0.570	0.153	0.078	0.453
Cysteine	11.9 ± 0.88	12.3 ± 0.88	0.718	0.844	0.651	0.025
α-Aminoadipic acid	14.0 ± 0.98	12.7 ± 0.98	0.326	0.125	0.158	0.045
Asparagine	5.26 ± 0.55	5.03 ± 0.53	0.501	0.006	0.230	0.002
Glutamine	4.32 ± 0.60	4.75 ± 0.60	0.468	0.735	0.068	0.665
Histidine	3.01 ± 0.43	3.42 ±0.43	0.513	0.677	0.033	0.256
Glycine	106 ± 12.9	98.0 ± 12.9	0.665	0.821	0.324	0.048
Arginine	23.0 ± 1.33	22.8 ± 1.33	0.864	0.349	0.051	0.107
τ-Methylhistidine	0.52 ± 0.05	0.54± 0.05	0.711	0.711	0.184	0.059
ß-alanine	4.99 ± 0.58	5.23 ± 0.58	0.410	0.068	0.549	0.611
γ-aminobutyric acid	1.98 ± 0.12	1.81 ± 0.12	0.013	0.060	0.487	0.766
α-Aminobutyric acid	2.01 ± 0.41	2.54 ± 0.51	0.055	0.368	0.763	0.105
Methionine	0.34 ± 0.04	0.29 ± 0.04	0.214	0.527	0.738	0.082
Phenylalanine	1.42 ± 0.16	1.35 ± 0.16	0.696	0.965	0.406	0.038
Leucine	4.41 ± 0.31	4.68 ± 0.33	0.561	0.842	0.079	0.885
Lysine	14.9 ± 0.98	15.1 ± 0.98	0.907	0.236	0.857	0.022
Proline	40.3 ± 2.65	40.8 ± 2.65	0.829	0.512	0.555	<0.001
Hydroxy proline	13.7 ± 0.98	13.1 ± 1.17	0.689	0.396	0.059	0.012
Total sum	1430 ± 80.8	1419 ± 80.8	0.848	0.623	0.061	0.363

EMMs, estimated marginal means; SE, standard error.

## Data Availability

As further project results are the subject of other publications that are currently under review, the data are available upon request from the co-author but are not yet stored in a repository.

## Supplementary Materials

The following supporting information can be downloaded at: https://www.mdpi.com/article/10.3390/ani15101414/s1, Supplementary Tables S1–S8.

## Abbreviations

The following abbreviations are used in this manuscript:

ABTS	2,2′-azino-bis(3-ethylbenzothiazoline-6-sulfonic acid)
AI	Atherogenic index
AlCl_3_	Aluminium chloride
BW	Body weight
*C*12:0	Lauric acid
*C*14:0	Myristic acid
*C*16:0	Palmitic acid
*C*18:0	Stearic acid
*C*18:1*cis*-11	*cis*- vaccenic acid
*C*18:2*cis*-9, trans-11	Rumenic acid
*C*18:4n-3	Stearidonic acid
C19:0	Nonadecanoic acid
*C*22:4n-6	Docosatetraenoic acid
*C*22:5n-3	Docosapentaenoic
CB1	Cannabinoid receptor 1
CBC	Cannabichromene
CBD	Cannabidiol
CBDA	Cannabidiolic acid
CBDV	Cannabidivarin
CBG	Cannabigerol
CBGA	Cannabigerolic acid
CBN	Cannabinol
CF	Crude fiber
*cis*-*C*18:1	Oleic acid
CON	Diet containing 3.5% soybean meal
CP	Crude protein
CV	Coefficient of variation
DM	Dry matter
DMI	Dry matter intake
DPPH	2,2-diphenyl-1-picrylhydrazyl
ECM	Energy corrected milk yield
EDTA	Ethylenediaminetetraacetic acid
EE	Ether extract
EMMs	Estimated marginal means
FAMEs	Fatty acid methyl esters
FRAP	Ferric-reducing antioxidant power
GABA	γ-aminobutyric acid
GC	Gas chromatography
h/H	Hypocholesterolemic/hypercholesterolemic ratio
HCl	Hydrogen chloride
HEMP	Diet containing 7.4% hemp leaves
HPI	Health-promoting index
HPLC	High-performance liquid chromatography
IL-1ß	Interleukin-1beta
K_2_CO_3_	Potassium carbonate
mBW	Metabolic body weight
ME	Metabolizable energy
MUFA	Monounsaturated fatty acid
Na_2_SO_4_	Sodium sulfate
PUFA	Polyunsaturated fatty acids
SFA	Sum of saturated fatty acids
SHB	Spent hemp biomass
TBARS	Thiobarbituric acid reactive substances
TEAC	Trolox-equivalent antioxidant capacity
TFA	Trans-fatty acids
THCA	Tetrahydrocannabinolic acid
TMR	Total mixed ration
TNF-α	Tumor necrosis factor alpha
TPTZ	2,4,6-Tri(2-pyridyl)-1,3,5-triazine
UFA	Unsaturated fatty acid
Δ8-THC	Δ8- tetrahydrocannabinol
Δ9-THC	Δ9-tetrahydrocannabinol
Δ9-THCV	Δ9-tetrahydrocannabivarin
