# Supplementary material for: Replacing Soybean Meal with Hemp Leaves in a Dairy Cow Diet: Plasma Antioxidative Capacity, Inflammatory Parameters and Milk Constituents
Source: Animals (Basel). 2025 May 14;15(10):1414. doi: 10.3390/ani15101414 (PMC12108515; doi:10.3390/ani15101414)
Supplement: Supplementary file 1 [file animals-15-01414-s001.zip › animals-3564760-supplementary.pdf]

Supplementary Material for:

Schwerdtfeger et al. Replacing soybean meal with hemp leaves in a dairy cow diet: plasma antioxidative capacity, inflammatory parameters and milk constituents

Supplementary Table S1. Primer sequences and PCR product sizes for genes analysed in buffy coat cells.

Gene	Function	Primer sequence (5′to 3′)	Accession ID	Size (bp)	Efficiency of amplification
YWHAZ	Forward	GAAAGGGATTGTGGACCAG	NM_174814.2	184	1.82
	Reverse	GGCTTCATCAAATGCTGTCT			
SDHA	Forward	TCCTGCAGACCCGGAGATAA	NM_174178.2	130	1.81
	Reverse	TCTGCATGTTGAGTCGCAGT			
RPS9	Forward	CGACCAAGAGCTGAAGCTGA	NM_001101152.2	161	1.81
	Reverse	GCAACAGGGCATTACCTTCG			
IL1B	Forward	GCTCTCCACCTCCTCTCACA	NM_174093.1	69	1.84
	Reverse	CTCTCCTTGCACAAAGCTCATG			
RELA	Forward	ATCTTTGACAACCGCGCC	NM_001080242	76	1.84
	Reverse	CGAGGCAGCTCCCAGAGTT			
TLR4	Forward	CTTGCGTACAGGTTGTTCCTAA	NM_174198	153	1.81
	Reverse	CTGGGAAGCTGGAGAAGTTATG			
TNF	Forward	CCTCTTCTCAAGCCTCAAGTAACAA	NM_173966.3	69	1.83
	Reverse	GCTGCCCCGGAGAGTT			

YWHAZ: tyrosine 3-monooxygenase/tryptophan 5-monooxygenase activation protein zeta; SDHA: succinate dehydrogenase complex flavoprotein subunit A; RPS9: ribosomal protein s9; IL1B: interleukin-1 beta; RELA: RELA proto-oncogene, NF-KB subunit; TLR4: toll like receptor 4; TNF: tumour necrosis factor

Supplementary Table S2: Feed constituents, nutrient composition and energy content of the diets (mean ± SD[1]).

Component, g/kg of DM	HEMP		CON		Wash-out diet	
	mean	SD	mean	SD	mean	SD
Grass-silage	201.05	56.67	208.17	57.70	222.51	42.69
Corn silage	395.57	47.22	410.47	51.18	346.54	32.11
Straw	--	--	17.71	0.29	11.88	7.30
Rapeseed extraction meal	87.13	6.76	77.66	9.65	67.47	14.72
Soybean extraction meal	--	--	35.06	3.11	--	--
Wheat seeds	24.00	7.86	24.84	7.97	21.88	8.90
Corn meal	61.24	6.24	63.54	6.81	54,31	6.00
Vicia faba	30.51	4.28	31.67	4.60	26.99	3.59
Lupin beans	15.89	11.28	16.54	11.75	13.52	9.82
Concentrate mix[2]	99.04	4.28	102.68	3.81	186.36	21.53
Mineral feed[3]	6.22	0.57	6.45	0.55	5.59	1.02
Hemp leaves	74.32	0.85	--	--	--	--
Limestone[4]	3.31	0.20	3.43	0.19	2.97	0.47
Soybean oil	1.72	0.19	1.78	0.19	1.55	0.31
Hay	--	--	--	--	8.43	8.47
Nutrients, g/kg of DM						
Crude ash	72.83	4.41	64.33	4.11	68.01	4.99
Crude protein	154.17	5.37	152.17	4.41	152.32	4.33
Crude fat	36.17	1.34	34.33	3.09	32.87	8.96
Crude fiber	161.33	4.71	173.33	17.33	171.18	7.20
ADFom	188.83	5.98	200.00	10.60	198.96	2.78
aNDFom	352.83	15.86	358.17	25.15	369.17	17.37
Starch	244.00	18.66	245.67	32.71	244.45	24.93
Secondary Plant components, mg/kg of DM						
Condensed tannins	4.52	3.63	4.14	3.48		
Total tannins	21.61	0.99	20.75	1.56		
Total phenols	50.93	7.58	46.99	6.24		
Total flavonoids	0.69	0.08	0.48	0.07		
DM and Energy						
DM, %	41.68	1.82	42.01	2.25	41.79	2.78
ME, MJ/kg DM	11.45	0.15	11.45	0.30	11.57	0.10
NEL, MJ/kg DM	7.02	0.11	6.97	0.21	7.09	0.11

SD: standard deviation, HEMP: diet containing 7.4% hemp leaves, CON: diet containing 3.5% soybean meal, DM: dry matter, ADFom: acid detergent fibre based on organic matter basis, aNDFom: neutral detergent fiber based on organic matter basis, ME: metabolisable energy, NEL: net energy of lactation

1 calculated from three blocks with each two periods

2 MF2000 (Ceravis Produktion und Transport GmbH, Malchin, Germany): composition: 24% crude protein, 2.6% crude fat, 5.1% crude fiber, 8% crude ash, 0.73% calcium, 0.5% phosphorus, 0.65% sodium, 7.1 MJ NEL/kg; Additives: 10,000 I.E. vitamin A, 1125 I.E. vitamin D3, 40 mg vitamin E, 0.6 mg I, 0.4 mg Co, 50 mg Mn, 75 mg Zn, 0.4 mg Se.

3 Panto Mineral R 8609 (HL Hamburger Leistungsfutter GmbH, Hamburg, Germany): composition: 20% calcium, 6% phosphorous, 8% sodium, 6% magnesium, 0.03% inorganic nitrogen, 13.7% phosphorous pentoxide.

Additives per kg original substance: 900,000 IU vitamin A, 200,000 IU vitamin D3, 4.5 g vitamin E, 1.5 g Cu, 8 g Zn, 5 g Mn, 60 mg I, 21 mg Co, 50 mg Se.

4 Bergophor $CaCO_3$ V001 (Hohburg Mineralfutter GmbH, Lossatal, Germany): 37% calcium

Supplementary Table S3: Cannabinoid concentration in hemp leaves of the variety 'Santhica 27'.

Cannabinoid	Concentration
CBDV, µg/kg	1462
CBD, mg/kg	52.3
Δ9-THCV, µg/kg	39.3
CBN, µg/kg	339
Δ9-THC, µg/kg	4958
Δ8-THC, µg/kg	< Lower detection limit*
THCA, µg/kg	2807

CBDV: cannabidivarin, CBD: cannabidiol, Δ9-THCV: delta-9-tetrahydrocannabivarin, CBN: cannabinol, Δ9-THC: delta-9-tetrahydrocannabinol, Δ8-THC: delta-8-tetrahydrocannabinol, THCA: tetrahydrocannabinolic acid, * Lower detection limit: 0.5 µg/kg

Supplementary Table S4: Percent of total fatty acids of hemp leaves, soybean meal, rapeseed meal and straw.

	Hemp leaves	Soybean meal	Rapeseed meal	Straw
**Saturated fatty acids (SFA)**				
C8:0	0.22	<0.01	<0.01	0.40
C10:0	0.07	<0.01	<0.01	0.30
C11:0	0.02	<0.01	<0.01	0.09
C12:0	0.10	0.08	<0.01	0.67
C13:0	<0.01	<0.01	<0.01	<0.01
C14:0	1.02	0.15	0.18	0.04
C15:0	0.22	0.07	0.14	0.63
C16:0	18.81	17.35	10.00	25.37
C17:0	0.24	0.14	0.11	0.96
C18:0	3.34	3.83	1.50	5.63
C20:0	2.42	0.29	0.37	3.64
C21:0	0.24	<0.01	<0.01	0.64
C22:0	2.69	0.36	0.32	3.67
C23:0	0.29	0.12	<0.01	1.31
C24:0	2.15	0.31	<0.01	3.58
C26:0	0.39	0.05	<0.01	3.50
Total SFA	32.22	22.79	13.33	50.44
**Monounsaturated fatty acids (MUFA)**				
C14:1cis-9	0.07	<0.01	<0.01	2.14
C16:1cis-9	0.77	0.25	1.13	0.64
C17:1cis-9	<0.01	<0.01	<0.01	<0.01
C18:1trans-9	0.07	0.03	0.95	0.04
C18:1trans-11	0.05	0.08	0.08	0.08
C18:1cis-9	2.08	16.45	39.97	13.30
C18:1cis-11	0.39	2.42	10.69	0.90
C20:1cis-11	0.17	0.03	<0.01	<0.01
C22:1cis-13	0.27	<0.01	<0.01	0.15
C24:1cis-15	<0.01	<0.01	<0.01	<0.01
Total MUFA	3.87	19.25	52.95	17.26
**Polyunsaturated fatty acids (PUFA)**				
C18:2trans	0.22	<0.01	<0.01	0.30
C18:2n-6 (LA)	9.37	50.46	27.40	20.22
C18:3n-6	0.87	0.05	<0.01	<0.01
C18:3n-3 (ALA)	51.71	7.25	5.93	10.13
C18:2cis-9,trans-11 (CLA)	<0.01	<0.01	<0.01	<0.01
C18:4n-3	1.38	0.03	<0.01	0.11
C20:2n-6	0.12	0.05	<0.01	0.03
C20:3n-9	<0.01	<0.01	<0.01	<0.01
C20:3n-6	<0.01	<0.01	<0.01	<0.01
C20:3n-3	<0.01	<0.01	<0.01	<0.01
C20:4n-6	<0.01	<0.01	<0.01	<0.01

C22:2n-6	<0.01	0.11	<0.01	<0.01
C20:5n-3(EPA)	<0.01	<0.01	<0.01	<0.01
C22:4n-6	<0.01	<0.01	<0.01	1.53
C22:5n-6	<0.01	<0.01	<0.01	<0.01
C22:5n-3 (DPA)	<0.01	<0.01	<0.01	<0.01
C22:6n-3 (DHA)	<0.01	<0.01	<0.01	<0.01
Total PUFA	63.66	57.96	33.72	32.31
n-6/n-3 ratio	0.20	6.93	4.55-	2.32

LA: linoleic acid, ALA: α-linolenic acid, CLA: conjugated linoleic acid, EPA: eicosapentaenoic acid, DPA: docosapentaenoic acid, DHA: docosahexaenoic acid

Supplementary Table S5: Concentrations of condensed tannins, total tannins and phenols and flavonoids in the hemp leaves, soybean meal, rapeseed meal and straw.

	**Hemp leaves**	**Soybean meal**	**Rapeseed meal**	**Straw**
Condensed tannins, mg/g DM	6.29	n.d.	n.d.	n.d.
Total tannins, mg/g DM	38.78	14.19	25.72	14.17
Phenols, mg/g DM	105.01	22.79	65.61	23.07
Flavonoids, mg/g DM	2.82	n.d.	n.d.	0.33
FRAP, mg/g DM	63.25	13.79	47.63	13.23
TEAC (ABTS), mmol/g DM	4.97	1.63	3.84	1.76
TEAC (DPPH), µmol/g DM	10.66	4.92	7.18	5.09

FRAP, ferric-reducing antioxidant power; TEAC, trolox equivalent antioxidant capacity determined by an ABTS, 2,2'-azino-bis(3-ethylbenzothiazoline-6-sulfonic acid) or DPPH, 1,1-diphenyl-2-picrylhydrazil assay; n.d.: not detected

Supplementary Table S6: Plasma free amino acid concentrations of cows fed a diet containing 7.4% Santhica 27 hemp leaves (HEMP, n = 12) or 3.5% soybean meal (CON, n = 12) on d 14 of the feeding period.

	EMMs ± SE		P-value			
**Amino acid, µM**	**HEMP**	**CON**	**Group**	**Period**	**Sequence**	**Block**
Aspartic acid	5.5 ± 0.25	5.4 ± 0.25	0.721	0.023	0.065	0.010
Glutamic acid	44.6± 1.91	47.0 ±1.91	0.371	0.863	0.660	0.895
Cysteine	27.1 ± 1.76	27.7 ± 1.76	0.526	0.837	0.762	0.463
α-Aminoadipic acid	4.8 ± 0.34	5.5 ± 0.34	0.039	0.298	0.444	0.367
Asparagine	47.2 ± 3.07	48.6 ± 3.07	0.721	0.279	0.546	0.315
Serine*	86.7 ± 5.21	82.7 ± 4.97	0.372	0.837	0.557	0.617
Glutamine	307 ± 18.8	313 ± 18.8	0.738	0.688	0.968	0.239
Histidine	47.1 ± 5.15	46.0 ± 5.15	0.775	0.304	0.658	0.064
Glycine*	424 ± 23.0	399 ± 21.6	0.133	0.128	0.330	0.849
Threonine	95.7 ± 8.2	104 ± 8.2	0.392	0.842	0.493	0.657
Citrulline	90.7 ± 3.9	90.7 ± 3.9	0.995	0.918	0.278	0.170
Arginine	82.3 ± 6.27	82.8 ± 6.27	0.943	0.071	0.646	0.305
π-Methylhistidine	3.9 ± 0.27	4.4 ± 0.27	0.002	0.042	0.029	0.550
τ-Methylhistidine	5.4 ± 0.19	5.4 ± 0.19	0.724	0.213	0.838	0.016
ß-alanine	4.341 ± 0.20	4.6 ± 0.20	0.126	0.164	0.912	0.482
Carnosin	23.6 ± 1.26	22.8 ± 1.26	0.657	0.652	0.249	0.580
Alanine*	251 ± 10.0	259 ± 10.0	0.445	0.279	0.235	0.019
Taurine	41.240 ± 2.52	47.3 ± 2.52	0.107	0.250	0.800	0.017
γ–aminobutyric acid & iso-aminobutyric acid	1.9 ± 0.08	1.8 ± 0.08	0.939	0.342	0.828	0.206
Anserine	0.8 ± 0.05	0.6 ± 0.05	<0.001	0.036	0.804	0.874
Tyrosine*	39.8 ± 3.8	40.2 ± 3.8	0.948	0.119	0.730	0.280
α-aminobutyric acid	16.8 ± 1.1	17.8 ± 1.1	0.515	0.904	0.063	0.767
Valine	226 ± 14	240 ± 14	0.258	0.530	0.589	0.280
Methionine*	23.1 ± 1.7	22.5 ± 1.6	0.757	0.196	0.980	0.193
Tryptophane	53.5 ± 2.43	49.9 ± 2.43	0.217	0.428	0.793	0.696
Phenylalanine	39.3 ± 2.63	40.0 ± 2.63	0.835	0.374	0.900	0.381
Isoleucine	107 ± 8.3	122 ± 8.3	0.056	0.238	0.622	0.404
Ornithine	34.4 ± 3.5	37.0 ± 3.5	0.440	0.871	0.984	0.361
Leucine	115.0 ± 9.3	119.0 ± 9.3	0.609	0.320	0.800	0.273
Lysine	57.6 ± 7.3	65.6 ± 7.3	0.137	0.147	0.971	0.302
Proline	130.0 ± 3.5	133.0 ± 3.5	0.488	0.180	0.937	<0.001

* Data was log transformed. Back-transformed data is shown for interpretation.
EMMs: estimated marginal means

Supplementary Table S7: Milk fatty acid profile (mg/100g) of cows fed a diet containing 7.4% Santhica 27 hemp leaves (HEMP, n = 12) or 3.5% soybean meal (CON, n = 12).

	EMMs ± SE		P-value			
	HEMP	CON	Group	Period	Sequence	Block
**Saturated fatty acids (SFA)**						
C6:0	1.40 ± 0.078	1.46 ± 0.078	0.490	0.212	0.942	0.765
C8:0	1.47 ± 0.057	1.50 ± 0.057	0.714	0.453	0.814	0.394
C10:0	3.33 ± 0.130	3.46 ± 0.130	0.389	0.325	0.565	0.325
C11:0	0.52 ± 0.028	0.55 ± 0.028	0.237	0.521	0.182	0.791
C12:0	4.00 ± 0.138	4.14 ± 0.138	0.374	0.266	0.923	0.460
C13:0	0.28 ± 0.017	0.28 ± 0.017	0.809	0.368	0.098	0.619
C14:0	12.7 ± 0.274	12.8 ± 0.274	0.683	0.953	0.481	0.954
C15:0	1.40 ± 0.072	1.30 ± 0.072	0.225	0.975	0.058	0.974
C16:0	31.0 ± 0.803	31.5 ± 0.803	0.291	0.024	0.527	0.611
C17:0	0.58 ± 0.020	0.53 ± 0.020	0.006	0.789	0.805	0.542
C18:0	9.17 ± 0.451	9.26 ± 0.451	0.825	0.134	0.461	0.571
C20:0	0.18 ± 0.007	0.17 ± 0.007	0.070	0.113	0.794	0.363
C21:0	0.06 ± 0.004	0.06 ± 0.004	0.379	0.788	0.252	0.273
C22:0	0.06 ± 0.004	0.05 ± 0.004	0.040	0.450	0.952	0.307
Sum SFA	66.3 ± 0.838	67.1 ± 0.838	0.276	0.860	0.862	0.905
**Monounsaturated fatty acids (MUFA)**						
C14:1cis-9	1.58 ± 0.077	1.65 ± 0.077	0.431	0.536	0.128	0.721
C16:1cis-9	2.15 ± 0.071	2.18 ± 0.071	0.751	0.481	0.022	0.876
C18:1trans-9	0.31 ± 0.008	0.31 ± 0.008	1.000	1.000	1.000	0.203
C18:1trans-11	1.14 ± 0.075	1.16 ± 0.075	0.863	0.035	0.885	0.692
C18:1cis-9	22.8 ± 0.709	22.3 ± 0.709	0.440	0.988	0.625	0.851
C18:1cis-11	1.48 ± 0.053	1.26 ± 0.053	0.011	0.495	0.646	0.525
C20:1cis-11	0.14 ± 0.004	0.12 ± 0.004	0.004	1.000	0.784	0.942
C22:1cis-13	0.07 ± 0.012	0.08 ± 0.012	0.141	0.442	0.327	0.343
Sum MUFA	29.7 ± 0.780	29.0 ± 0.780	0.398	0.942	0.937	0.892
**Polyunsaturated fatty acids (PUFA)**						
C18:2n-6 (LA)	2.17 ± 0.058	2.08 ± 0.058	0.239	0.210	0.015	0.394
C18:3n-6	0.03 ± 0.002	0.03 ± 0.002	0.563	0.563	0.664	0.333
C18:3n-3 (LNA)	0.59 ± 0.015	0.46 ± 0.015	<0.001	0.428	0.045	0.178
C18:2cis-9.trans-11 (CLA)	0.54 ± 0.016	0.61 ± 0.016	0.004	0.880	0.096	0.001
C18:4n-3	0.10 ± 0.006	0.10 ± 0.006	0.418	1.000	0.042	0.735
C20:2n-6	0.07 ± 0.005	0.06 ± 0.005	0.600	0.135	0.241	0.283
C20:3n-6	0.13 ± 0.007	0.12 ± 0.007	0.304	0.880	0.597	0.099
C20:4n-6	0.13 ± 0.004	0.12 ± 0.004	0.001	0.624	0.242	0.164
C22:2n-6	0.04 ± 0.006	0.04 ± 0.006	0.894	0.034	0.097	0.648
C20:5n-3(EPA)	0.04 ± 0.003	0.04 ± 0.003	0.150	0.828	0.865	0.350
C22:4n-6	0.13 ± 0.017	0.13 ± 0.017	0.784	0.030	0.069	0.440
C22:5n-6	0.12 ± 0.017	0.09 ± 0.017	0.071	0.408	0.123	0.152
Sum PUFA	4.08 ± 0.100	3.88 ± 0.100	0.071	0.468	0.373	0.581

Supplementary Table S8: Whey free amino acid concentrations of cows fed a diet containing 7.4% Santhica 27 hemp leaves (HEMP, n = 12) or 3.5% soybean meal (CON, n = 12) in pooled milk samples collected on d 11 and 12 of the feeding period.

	**EMMs ± SE**		***P*-value**			
**Amino acid, µmol/l**	**HEMP**	**CON**	**Group**	**Period**	**Sequence**	**Block**
Arginine	23.0 ± 1.33	22.8 ± 1.33	0.864	0.349	0.051	0.107
Glutamine	4.32 ± 0.60	4.75 ± 0.60	0.468	0.735	0.068	0.665
Glutamic acid	543 ± 46.2	554 ± 46.2	0.570	0.153	0.078	0.453
Cysteine	11.9 ± 0.88	12.3 ± 0.88	0.718	0.844	0.651	0.025
α-Aminoadipic acid	14.0 ± 0.98	12.7 ± 0.98	0.326	0.125	0.158	0.045
Asparagine	5.26 ± 0.55	5.03 ± 0.53	0.501	0.006	0.230	0.002
Histidine	3.01 ± 0.43	3.42 ±0.43	0.513	0.677	0.033	0.256
Serine	13.8 ± 1.44	13.5 ± 1.41	0.799	0.831	0.103	0.107
Glycine	106 ± 12.9	98.0 ± 12.9	0.665	0.821	0.324	0.048
iso-Aminobutyric acid	0.37 ± 0.04	0.42 ± 0.04	0.124	0.588	0.825	0.484
Threonine	445 ± 28.4	429 ± 28.4	0.525	0.588	0.109	0.418
Tyrosine	2.30 ± 0.22	2.30 ± 0.22	0.998	0.989	0.295	0.195
π-Methylhistidine	2.02 ± 0.35	2.74 ± 0.35	0.120	0.583	0.246	0.379
τ-Methylhistidine	0.52 ± 0.05	0.54± 0.05	0.711	0.711	0.184	0.059
ß-alanine	4.99 ± 0.58	5.23 ± 0.58	0.410	0.068	0.549	0.611
Carnosin	3.18 ± 0.40	3.23 ± 0.40	0.842	0.643	0.302	0.599
Alanine	57.6 ± 3.06	56.6 ± 3.06	0.740	0.177	0.126	0.264
Taurine	37.6 ± 4.53	38.4 ± 4.53	0.762	0.139	0.765	0.427
γ-aminobutyric acid	1.98 ± 0.12	1.81 ± 0.12	0.013	0.060	0.487	0.766
Anserine	0.35 ± 0.04	0.33 ± 0.04	0.485	0.177	0.657	0.327
α-Aminobutyric acid	2.01 ± 0.41	2.54 ± 0.51	0.055	0.368	0.763	0.105
Valine	13.8 ± 0.97	14.5 ± 0.97	0.188	0.291	0.137	0.837
Methionine	0.34 ± 0.04	0.29 ± 0.04	0.214	0.527	0.738	0.082
Tryptophane	1.89 ± 0.22	1.66 ± 0.22	0.409	0.513	0.148	0.232
Phenylalanine	1.42 ± 0.16	1.35 ± 0.16	0.696	0.965	0.406	0.038
Isoleucine	5.33 ± 0.45	5.86 ± 0.45	0.218	0.181	0.223	0.667
Ornithine	4.22 ± 0.50	4.79 ± 0.50	0.170	0.528	0.754	0.766
Leucine	4.41 ± 0.31	4.68 ± 0.33	0.561	0.842	0.079	0.885
Lysine	14.9 ± 0.98	15.1 ± 0.98	0.907	0.236	0.857	0.022
Hydroxy proline	13.7 ± 0.98	13.1 ± 1.17	0.689	0.396	0.059	0.012
Proline	40.3 ± 2.65	40.8 ± 2.65	0.829	0.512	0.555	<0.001
Total sum, mmol/l	1.43 ± 80.8	1.42 ± 80.8	0.848	0.623	0.061	0.363
